# Systematic Review of AI‐Driven Applications for Screening Nasopharyngeal Carcinoma Using MRI

**DOI:** 10.1002/jmri.70445

**Published:** 2026-07-26

**Authors:** Lun M. Wong, Ho Sang Leung, Zuyao Yang, Yip Man Tsang, Yuet Ting Chan, Tiffany Y. So, Ann D. King, Qi Yong H. Ai

**Affiliations:** ^1^ Department of Imaging and Interventional Radiology Faculty of Medicine, The Chinese University of Hong Kong, Prince of Wales Hospital Hong Kong China; ^2^ The CU Lab of AI in Radiology, Department of Imaging and Interventional Radiology Faculty of Medicine, The Chinese University of Hong Kong, Prince of Wales Hospital Hong Kong China; ^3^ Department of Imaging and Interventional Radiology Prince of Wales Hospital, Hospital Authority Hong Kong China; ^4^ JC School of Public Health and Primary Care, The Chinese University of Hong Kong Hong Kong China

**Keywords:** artificial intelligence, benign hyperplasia, deep learning, intravenous contrast, magnetic resonance imaging, nasopharyngeal carcinoma, screening

## Abstract

**Background:**

Nasopharyngeal carcinoma (NPC) can be detected early on MRI, but adoption for screening is limited by a shortage of experienced specialists. MRI artificial intelligence (AI) algorithms for diagnosing NPC in the literature may address this, but most studies focus on routine clinical care, and their applicability in screening requires close examination.

**Purpose:**

To (i) evaluate the diagnostic performance of AI for NPC detection, (ii) analyze the impact of study protocols, and (iii) assess the applicability of existing studies to NPC screening.

**Study Type:**

Systematic review.

**Population:**

Thirty‐eight studies were included, including a total of 23,398 patients (20,693 NPC and 2705 non‐NPC).

**Fieldstrength:**

1.5 T to 3.0 T.

**Assessment:**

Following PRISMA guidelines, PubMed, Scopus, and Embase records from January 1, 2009 to August 16, 2025 were screened by two independent reviewers for studies on NPC detection, localization, and/or diagnosis using MRI. Included studies were reviewed for applicability and risk of bias. Lesion‐localization performance (Dice similarity score [DSC]) and NPC vs. non‐NPC discrimination (sensitivity/specificity) were extracted and summarized. Subgroup analyses assessed the impact of intravenous contrast on AI performance.

**Statistical Tests:**

Meta‐analysis used standard random‐effects univariate restricted maximum likelihood model and hierarchical summary receiver operator characteristics for performance pooling and subgroup analysis, taking *p* value < 0.05 as significance.

**Results:**

Pooled performance from 30 localization and 6 discrimination studies was DSC = 0.80 (CI: 0.78–0.82) and sensitivity/specificity = 97.1% (CI: 88.0%–99.3%)/87.8% (CI: 78.9%–93.2%), respectively. Intravenous contrast did not significantly affect AI performance for either task (*p* > 0.05). Only seven studies were fully applicable to screening.

**Data Conclusion:**

AI‐driven NPC screening using MRI is feasible at high sensitivity. Using non‐contrast MRI did not significantly impact AIs' performance. However, applicability and the suboptimal ratio of NPC: non‐NPC patients remain the weaknesses of existing studies, warranting further investigation with cohorts that better reflect the screening scenario.

**Systematic Review Registration:**

The systematic review protocol of this system is registered with the Inplasy registry (Ref. INPLASY202570076).

**Abbreviations**
AI: Artificial intelligence
ASD: Average surface distance
AUC: Area under the curve
CI: Confidence interval
CLAIM: Checklist for AI in Medical Imaging
DL: Deep learning
DSC: Dice similarity score
EBV: Epstein–Barr Virus
HSROC: Hierarchical Summary Receiver Operator Characteristics
IoU: Intersection‐over‐union
IQR: Inter‐quartile range
NP: Nasopharynx
NPC: Nasopharyngeal carcinoma
PPV: Positive predictive value
QUADAS: QUality Assessment of Diagnostic Accuracy Studies
REML: Restricted maximum likelihood model
ROI: Region‐of‐interest

## 1 Background

Undifferentiated nasopharyngeal carcinoma (NPC), which is endemic in countries and regions around the Southeast China Sea, significantly affects middle‐aged men who are in the prime of their lives. NPC has been consistently the most common cancer in men aged between 20 and 44 for the past 15 years in Hong Kong [1], and ranks among the top‐three cancers in men aged 30–49 in Singapore between 2017 and 2022 [2]. NPC is curable at an early stage (stage I–II) and is treated with intensity modulated radiotherapy only, with a high 5‐year recurrence‐free survival rate [3]. Unfortunately, early NPC is typically asymptomatic and goes undetected so that by the time of clinical presentation, over 70% of new NPC patients have advanced stage disease [1]. Despite treatment with chemoradiotherapy, which has a significantly higher treatment toxicity than radiotherapy alone, outcomes and survival are lower than those with early‐stage disease.

Recently, there have been major breakthroughs in NPC screening using Epstein–Barr Virus (EBV)‐related serological tests, including antibodies and plasma DNA, that showed a high sensitivity for detecting NPC [4, 5, 6, 7], which includes early NPCs. Recent recommendations have suggested potential benefits for these screen‐positive subjects, who still have a notable rate of false positives, to undergo a fast, non‐contrast‐enhanced MRI prior to endoscopy that might prioritize suspicious patients and guide subsequent biopsy [8]. MRI offers a more comprehensive internal view of the nasopharynx (NP) than endoscopy, and contrast‐enhanced images have been shown to detect more cancers than endoscopic examination, up to 10% and 17%–34% more, in symptomatic and asymptomatic screened patients, respectively, most of which are early NPCs [9, 10, 11, 12]. Cost remains a hurdle for the cost‐effective implementation of an MRI‐based screening program, so with this in mind, and with the desire to avoid injection of a contrast agent, recent work has shown the value of a fast, non‐contrast‐enhanced MRI for screening purposes [13]. However, non‐contrast MRI assessment for NPC screening is challenging, requiring a high level of radiology expertise for interpretation, which is not widely available and can be expensive.

In this regard, the swift advancement of artificial intelligence (AI) not only promises a more cost‐effective but also more accessible and quality‐consistent assessment for detecting NPC on MRI. In particular, an AI‐driven algorithm to automatically assess the NP, detect and segment abnormalities, and then characterize them to discriminate NPC from benign changes will substantially facilitate the realization of a cost‐effective screening program, especially in individuals with high risk of developing NPC identified through preliminary means including EBV DNA screening. Nevertheless, while there has been a sizable body of AI studies reporting algorithms that aid the MRI management of NPC, few of them [14, 15, 16] discussed their applications in the context of NPC screening despite its importance to the outcome of patients in endemic regions.

In particular, the evaluation of AI‐driven algorithms for screening purposes presents unique challenges that are distinct from standard diagnostic workflows, especially regarding the tested data demographics and the imaging modalities used [17]. More specifically, NPC screening in asymptomatic populations anticipates the majority of participants to test negative for NPC, which imposes limitations on the imaging protocol. Given that existing AI studies are often conducted using arbitrary scanning protocols, further adaptation of AI for screening MRI necessitates a thorough understanding of the potential impacts of these data domain shifts on AI performance, which is an area that has not been investigated. In particular, previous literature reviews and meta‐analyses of AI applications for NPC management [18, 19, 20, 21] have not focused on NPC screening or early detection.

Therefore, this systematic review and meta‐analysis aims to answer three critical questions. First, what is the reported performance for AI NPC localization and for discrimination of NPC from non‐NPC lesions on MRI? Second, to what extent do screening‐relevant clinical constraints, such as the adoption of suitable MRI protocols and the characteristics of the patient population, influence the performance of AI systems? Third, how applicable are existing AI studies to the screening of NPC on MRI?

## 2 Materials and Methods

### 2.1 Literature Search

#### 2.1.1 Inclusion Criteria and Eligibility

The review covers all original study articles that proposed and evaluated AI‐driven algorithms that have the potential to facilitate early detection of NPC on MRI. Specifically, this review includes studies that used AI or radiomics for either detection, segmentation, or discrimination of NP lesions with a diagnosis focus. Studies investigating post‐diagnosis steps, or conducted in non‐endemic regions, were excluded. Only peer‐reviewed journal articles reporting original studies written in English were eligible for inclusion. Detailed inclusion criteria and eligibility descriptions were available in the Supporting Information.

#### 2.1.2 Literature Search Protocol

Publications within our review scope were searched systematically from within three established registries: PubMed, Scopus, and Embase. Based on the inclusion criteria, the articles were published between 1st January 2009 and 16th of August 2025. Search was conducted using the following inclusion keywords: MRI, NPC, screening, detection, segmentation, discrimination, radiomics, deep learning, artificial intelligence, and neural network. The exact search terms for each database are tabulated in Table S1. This protocol has been registered under the Inplasy registry (Ref. INPLASY202570076).

### 2.2 Inclusion Decision

After the initial search results were collected, duplicated records were first removed based on comparison of title, publisher and digital object identifiers automatically. Unique publications were then preliminarily screened manually based on their meta‐data, title, abstract and keywords to exclude articles that were not eligible for inclusion. The remaining articles were manually inspected in full text by two reviewers (L.M.W. & Q.Y.H.A.) independently for inclusion. Conflicts regarding inclusion decisions between the two reviewers were resolved by a third reviewer, who is a board‐certified radiologist (H.S.L.) subspecialized in head and neck imaging. Reference lists of included articles were cross‐checked for articles that were potentially missed, these articles were included based on consensus among all three reviewers.

The Preferred Reporting Items for Systematic Review and Meta‐Analyses (PRISMA) guideline was followed for this process and a PRISMA flow chart was plotted.

### 2.3 Systematic Review

#### 2.3.1 Risk of Bias and Applicability Concerns Evaluation

QUality Assessment of Diagnostic Accuracy Studies (QUADAS)‐2 tool [22] was used for evaluating the risk of bias of the reviewed studies, and signaling questions were designed based on the components in the Checklist for Artificial Intelligence in Medical Imaging (CLAIM) checklist [23]. The individual answers corresponding to the CLAIM items were aggregated as “Low”, “High”, or “Unclear” for each of the four QUADAS‐2 domains, and then into a three‐level risk of bias rating of “Low”, “Med” and “High” for the article. For applicability concern, we curated a checklist corresponding to the QUADAS‐2 “Patient Selection” domain (Table 1) corresponding to screening NPC. Aligned with QUADAS‐2, items were rated into “Yes”, “No” or “Unclear”, and the applicability concerns were then aggregated into a “Low”, “High” and “Unclear” ratings.

**TABLE 1:** Items for evaluating the applicability and pertinence of the studied data for nasopharyngeal carcinoma (NPC) screening.

Applicability concern evaluation form
Studied Population
□ Included NPC patient population include stage T1 patients □ Included non‐NPC with normal nasopharynx or benign lesions in the nasopharynx □ Among included NPC patients, distribution of early(I/II):advance(III/IV) is close to 7:3 MRI Protocol □ MRI protocol required by the AI does not involve the use of intravenous contrast □ AI requires only one single MRI sequence

Two independent reviewers (L.M.W. & Q.Y.A.) evaluated the risk of bias and applicability concern for each study based on the signaling questions and checklist items. Differences in decisions were resolved by consensus. Additional details, including the mapping between CLAIM checklist items to QUADAS‐2 domains and requirements for reference standard are given in Table S2. It should be noted that, unless an “unclear” judgment for “patient selection” (risk of bias) is reached (which may also lead to “unclear” applicability for some of the signaling questions), the two types of concern judgments are otherwise distinct and are not overlapping or double‐counted.

### 2.4 Data Extraction

Data were systematically extracted from the included articles in accordance with a predefined extraction guideline (Table 2). The studies were categorized based on the task nature of their proposed AI algorithms into one of three groups: (i) region‐of‐interest (ROI) detection, (ii) segmentation, and (iii) lesion discrimination, with (i) & (ii) corresponding to localization and (iii) corresponding to discrimination between NPC and non‐NPC at the per‐patient level. For each task, relevant performance metrics were extracted alongside essential information such as sample size, patient characteristics, validation framework, and the MRI sequences utilized by the AI models. To address our core research questions, we further investigated whether the proposed AI algorithms incorporated multiple‐sequence and/or contrast‐enhanced sequence inputs, which are two critical factors pertinent to screening.

**TABLE 2:** Data extraction protocol.

Category	Data field	Values	Definitions/Details
Study Design	Validation framework	All	All included data were used in both training and testing
		Train‐test	Included data was split into training and testing cohorts.
		Train‐test‐validation	Included data was split into training, validation and testing cohorts.
		k‐fold	Included data were involved in K‐fold cross‐validation
		External	Validation was conducted over external dataset
Patients	Number of patients included	—	The number of all patients included in the study
	Validation sample size	—	The number of patients used for validation, this depends on the study design: ‐ If “all”, it's size of entire cohort. ‐ If ‘external’, we prioritized using the sample size of the external cohort. ‐ If ‘train‐test’ or ‘train‐test‐validation’, testing cohort size. ‐ If ‘k‐fold’, we used all patients involved in the k‐fold cross‐validation.
AI's characteristics^a^	Type of AI task (multiple selection)	ROI detection	AI aims to detect the position of NP lesion by labelling a rectangular ROI (2D/3D)
		Segmentation	AI aims to delineate the contour of NP lesion
		Discrimination	AI aims to characterize a lesion in the NP (with or without prior knowledge of existence of a lesion)
	Sequence used (multiple selection)	T1w	T1‐weighted images
		T2w	T2‐weighted images
		CE	Prefix for contrast enhanced images
		FS	Suffix for fat‐suppression
		Others	Other sequences including functional MRI.
	Multi‐sequence	Yes	AI requires multiple sequences as input
		No	AI can operate on only one sequence as input
	Dimensionality	2D	AI requires 2D input
		3D	AI requires 3D input
		Pseudo 3D	AI requires 3D input but processes it slice‐by‐slice before aggregating results back into 3D. If it does not aggregate results back into 3D, it's categorized as 2D algorithm.
AI's performance^b^	Rectangular ROI detection	IoU	Intersection over union
		Sensitivity	Sensitivity of detecting a lesion
	Segmentation	DSC	Dice similarity coefficient
		ASD	Average surface distance (mm)
	Discrimination	Sensitivity	Sensitivity of identifying NPC versus non‐NPCs
		Specificity	Specificity of identifying NPC versus non‐NPCs
		AUC	Area under the receiver operator characteristics

*Note:* Key information for meta‐analysis was extracted using this guideline.

Abbreviations: AI, artificial intelligence; NP, nasopharynx; ROI, region‐of‐interest.

^a^ Prioritized to report the characteristics of models concluded by the original authors. If the original authors did not conclude the recommendation of any model, the model with the best overall performance was reported.

^b^ Prioritize reporting performance of models on the external data, reporting the performance of the models recommended by the authors. If they did not conclude the recommendation for any model, the model with the best overall performance was reported.

### 2.5 Meta‐Analysis for Assessing AI's Performance

Meta‐analysis was performed to pool the performance of AI for the three defined categories. Additionally, moderator and sub‐group analyses were performed to understand if applicability requirements, in particular the avoidance of using contrast‐enhanced MRI and using a short protocol of only one MRI sequence, were significant factors that could have affected the AIs' performance.

#### 2.5.1 Localization Accuracy Metrics

We pooled the performance reported by detection and segmentation studies, including intersection‐over‐union (IoU), Dice similarity coefficient (DSC), and average surface distance (ASD), as single‐group measures to synthesize the achievable performance for evaluated tasks. The pooled performance was estimated by fitting the extracted means, SD, and testing sample size using a univariate restricted maximum likelihood model (REML) with random effects to account for inter‐study variations [24].

#### 2.5.2 Diagnostic Test Metrics

Diagnostic test metrics were primarily used in the discrimination task. As the extracted sensitivity and specificity are correlated variables, we used Hierarchical Summary Receiver Operating Characteristics (HSROC) model to pool the performance, which takes into account the trade‐off relationship between these two metrics using bivariate modeling [25].

#### 2.5.3 Heterogeneity Analysis

Heterogeneity was estimated with Cochran's Q, Higgins *I* ^*2*^ and $\tau^{2}$, performed with sample size adjustment based on Holling's method where appropriate [26], which represent the proportion of total observed variation across studies that is due to true differences in effects rather than chance, and the absolute variance of the true effect sizes underlying those studies, respectively.

#### 2.5.4 Sensitivity Analysis

Sensitivity analysis was conducted by repeating the meta‐analysis in a leave‐one‐out fashion. This approach involved iteratively excluding one study at a time to assess its impact on the overall heterogeneity measures and the pooled mean as well as the confidence interval. Studies that exhibited a significant shift in the pooled results were excluded. Where the potential for significant overlap in datasets between studies was detected, we performed multi‐drop sensitivity analysis, iteratively dropping one study from each overlapping pair for all combinations to evaluate their impact on the pooled performance.

### 2.6 Subgroup Analysis of AIs' Performance

To understand how constraints of screening application will affect performance, we additionally performed meta‐analysis with moderators and subgroup analysis corresponding to the use of only non‐contrast MRI scans and only a single MRI sequence. Studies that did not clearly report these details were further excluded. Additionally, we grouped studies that utilized deep learning algorithms for comparison to radiomics with conventional machine learning algorithms to investigate any differences.

### 2.7 Missing Value Handling

For single group performance metrics, missing means or SDs were estimated based on median or inter‐quartile range (IQR) if reported [27], or imputed with simple regression based on observed values including sample sizes, means, SDs, and 95% confidence intervals (CI) of the collected metrics reported by other reviewed studies. For diagnostic test metrics, missing confusion matrix counts (TP, TN, FP, and FN) were computed from the number of patients, their characteristics, and the AI's sensitivity and specificity reported. Studies that did not report sensitivity and specificity were omitted.

### 2.8 Statistical Analysis Software

All statistical analysis was performed using R programming language, primarily utilizing two libraries including “metafor” (version 4.6‐0) [28] and “mada” (version 0.5.11) [29]. Missing data imputation was conducted using Python's “scikit‐learn” package (version 1.5.1) [30].

## 3 Results

### 3.1 Included Articles

**FIGURE 1:**
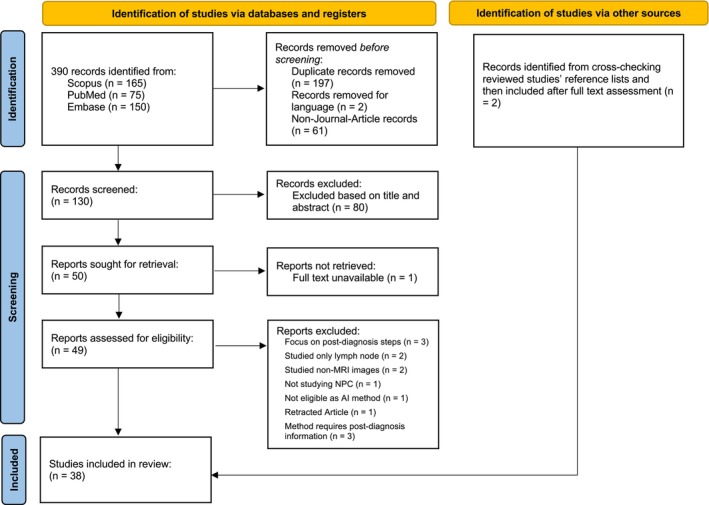
Flowchart of systematic review of papers inclusion following PRISMA guideline.

A total of 390 articles were identified from the three databases using the listed search terms for papers published between 1st Jan 2009 to 16th August 2025, of which 197 duplicated entries, 61 non‐original articles, and 2 non‐English entries were dropped using automatic tools. Subsequently, 80 studies were excluded after manually reviewing the abstract and title. The remaining 50 articles were sought for retrieval for full‐text examination. Except for one article which full text was unavailable, the rest 49 articles were independently examined by two reviewers for inclusion. One more article was found to have been retracted and 2 articles were additionally assessed through cross‐referencing. Finally, 38 articles were included in the analysis. These 38 studies assessed a total of 23,398 patients (20,693 NPC and 2705 non‐NPC). A flowchart following the recommendation of PRISMA is provided in Figure 1.

### 3.2 Studies Overview

**FIGURE 2:**
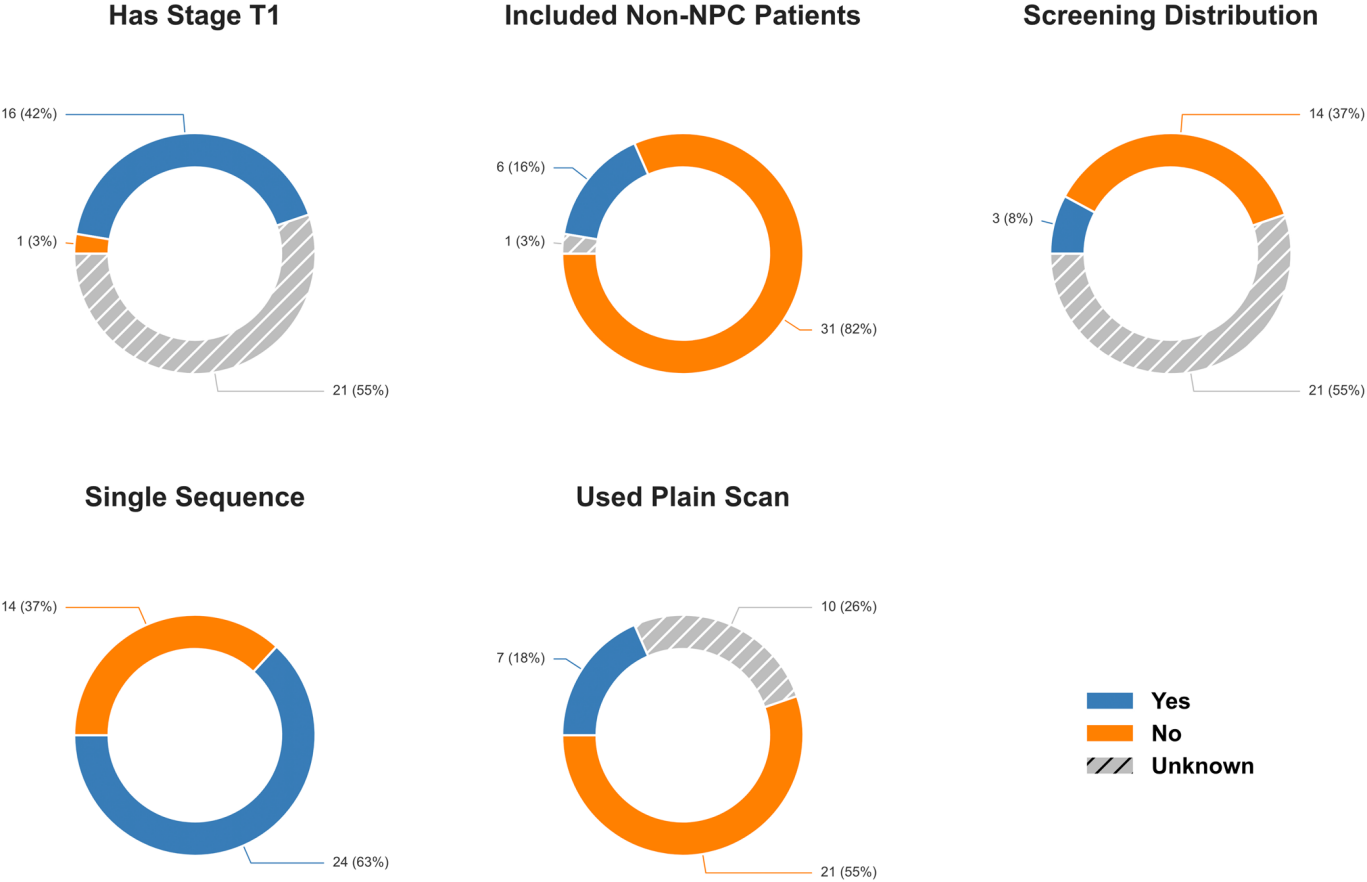
Pie charts illustrating the evaluation results for NPC screening applicability criteria. “Unknown” indicates that the information was not available for extraction from the article, which also led to an ‘unclear’ judgment for the corresponding applicability signaling question. NPC = nasopharyngeal carcinoma; Non‐NPC = patients without NPC.

**FIGURE 3:**
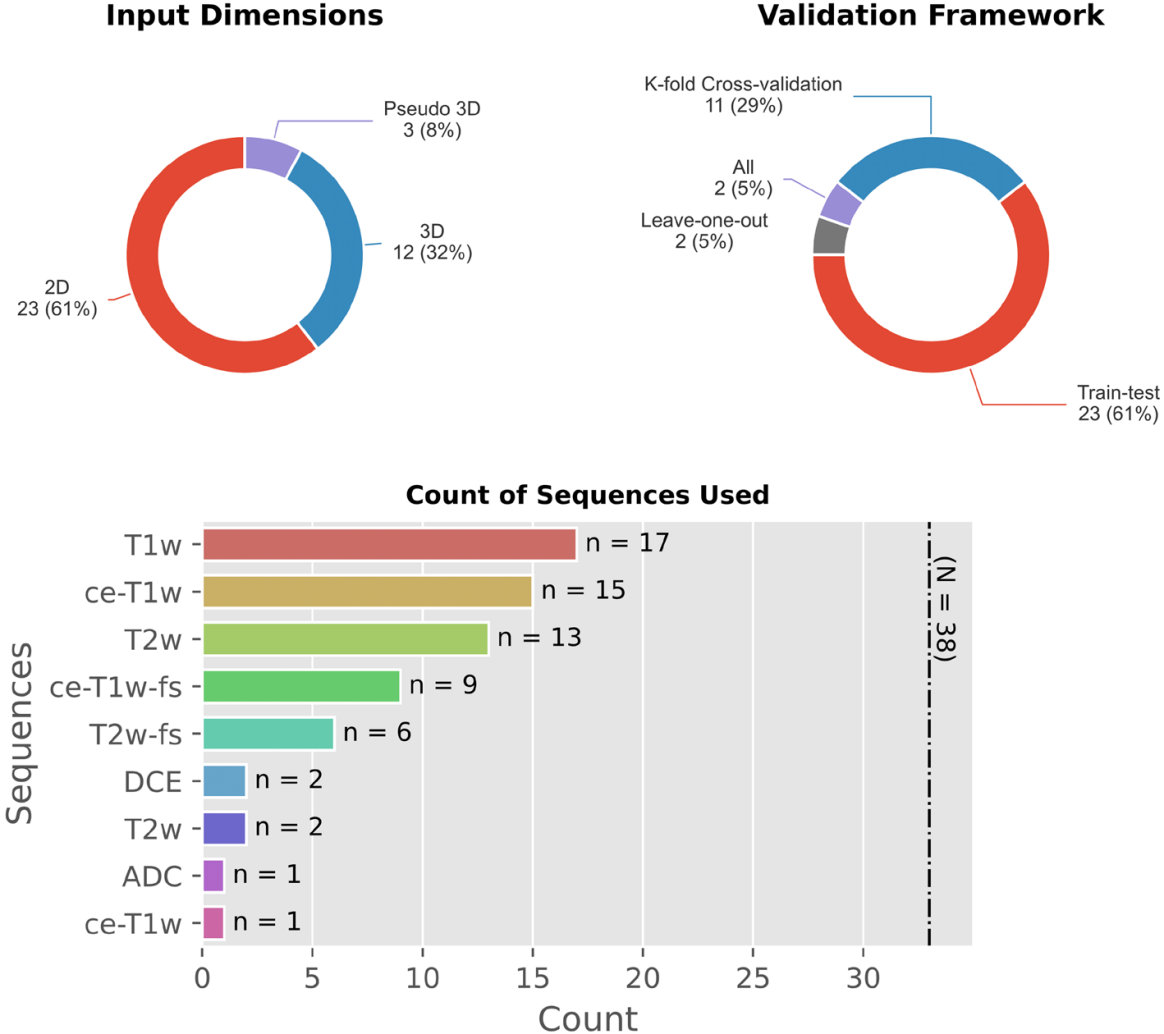
Summary of AI configurations and validation framework of the 33 reviewed studies. Input dimension indicates how the images were prepared prior to input, 3D indicates either the entire volume or a sub‐volume was served as input, 2D indicates the AI was based on slices of input, pseudo 3D indicates the AI required 3D input but processed it in a 2D‐like manner. For validation framework, “All” suggest the study used the entire dataset for both training and validation. ce‐T1w‐fs = contrast‐enhanced T1‐weighted fat‐suppressed, T2w = T2‐weighted, DCE = dynamic contrast‐enhanced, ADC = apparent diffusion coefficient map.

All studies were conducted with a retrospective patient cohort; the majority proposed and evaluated deep learning algorithms (*n* = 35), while the rest studied conventional machine learning (*n* = 3). A summary of the methodologies including the used patient cohort, MRI sequence, the input configuration, and the validation framework is given in Figures 2, 3. Additionally, we have reviewed in‐depth studies with low applicability concerns, a summary of their methodology, purpose, strengths and weaknesses (Table 3).

**TABLE 3:** Summary of methodology strengths and weaknesses of studies highly applicable to screening NPC (low applicability concern).

References #	Year	Patients(NPC vs non‐NPC T1/T2/T3/T4)	Name of network(s)/method(s)*	MRI sequence(s)*	Key methodology	Key conclusion and contribution	Additional queries	Conclusion from additional queries	Notable limitations
[14]	2020	3142 vs. 958 739/297/1547/559	SC‐DenseNet	ce‐T1w	Shared DenseNet encoder for segmentation decoder and discrimination output layer for guaranteed association between segmentation and NPC/non‐NPC prediction. Network was constrained to generate primary NPC segmentation only when output discrimination prediction is +ve of NPC.	Novel network design and training strategy to train a model with shared encoder for segmentation and discrimination. One of the studies with the largest dataset > 4000 cases with substantial heterogeneity that can represent better real‐life scenario.	Performance compared to radiologist: Compared performance with 3 radiologists in terms of AUC. Segmentation performance across T‐stage: ANOVA was conducted to evaluate the DSC across different T‐stages.	Proposed network showed higher AUC (0.956) than all three observers (0.912–0.930), no statistical test was performed for this comparison. Proposed network showed no differences in DSC between different T‐stages (all *p* > 0.05). Distribution of DSC across T‐stage showed no significant differences (x^2 = 0.275, *p* = 0.069). [Note: Chi‐square was reported ANOVA was reported in methods.]	Single‐center without external validation. Results generated from a single data‐split that can subject to selection bias. Statistical analysis was conducted mainly for segmentation (i.e., DSC) but not discrimination. No direct comparison between radiologists and AI. These factors led to “medium” risk of bias judgment for this article.
[16]	2021	203 vs. 209 203/0/0/0	SW‐RAN	T2w‐fs	Slicewise‐attention mechanism to account for different importance of context in each slice for end‐to‐end binary classification into NPC and non‐NPC.	One of the first studies to investigate non‐contrast MRI for discriminating between NPC and benign hyperplasia Focused on the most challenging group (stage T1 NPC) that is more commonly encountered during screening. Statistical comparison with radiologist who devised a plain‐scan grading system Accounted biopsy prior to scan, a major confounding factors for retrospective analysis, with NPC often confirmed through biopsy whilst not always the case for non‐NPC patients.	Performance compared to radiologist: Compared performance with radiologists using Chi‐square test. Was prior biopsy a confounding factor: Multi‐variate logistic regression to investigate if NP biopsy prior to MRI, performed in most NPC patients but not in non‐NPC patients, were a significant factor compared to DL prediction.	DL performance was comparable to a senior radiologist looking at T2w‐fs only (χ^2 = 3.3, *p* = 0.07). Biopsy was not a significant predictor when adjusted by DL prediction (*p* = 0.84)	Single‐center without external validation.
[31]	2022	1823 vs. 663 ?	NP Localization: Deeplabv3 + Unet Discrimination: EfficientNet‐B0/ResNet34/MobileNetv3‐Large/DenseNet121	T2w	Locate NP region by formulating the problem as segmentation of the entire NP region, which center‐of‐masses were used to crop 2D square patches, which were pipelined for discrimination by 4 different DL models, each trained for multi‐class classification into either of NPC, LH, lymphoma, chordoma, craniopharyngioma or normal class.	Included patients non‐NPC with other head and neck abnormalities and performs multi‐class classification for these diseases as well. In‐depth investigation of network configurations that covers input format, model architectures and interpretability of models.	Interpretability of trained networks: Manual inspection of Grad‐CAM output by 3 reviewers who rate the interpretability into score 0 (worst) to 1 (best) with 5 score levels. Required training dataset volume: Evaluate performance when trained on 25%, 50% and 100% of training data. Optimal input size for discrimination: Evaluated performance when input is cropped to different sizes (112px, 224px and 512px) of square patches	The size input 112px presents best interpretability potential (mean score: 0.735 ± 0.097). No between‐model comparison results reported. Using 50% (~933 patients, 4989 slices), 25% training set (~467 patients, 2495 slices) yield adequate performance, with best one‐vs‐rest AUC for NPC of 0.96 and 0.90. Optimal size for input is 112px square patches, with both better performance and better interpretability.	Patient inclusion/exclusion criteria was vague, and the reference standard for “normal” and “diseased” were defined on a slice‐level rather than patient‐level. Furthermore, patients' clinical information, such as T‐stage distribution, was not clearly given. These factors led to “medium” risk of bias for this article. Single‐center without external validation. Results generated from a single data‐split that can subject to selection bias. A final recommendation of model and hyperparameter was not given.
[32]	2022	3582 vs. 896 802/364/1776/640	SC‐DenseNet/V‐Net	T1w/T2w/ce‐T1w	Retrained SC‐DenseNet with a larger group of patient on 3 sequences, and compare the plain scan results with ce‐T1w results.	One of the studies with the largest dataset > 4000 cases with substantial heterogeneity that can represent better real‐life scenario. Evaluated and compared performance of AI models on plain scan and ce‐MRI.	Behavior of another network: Study was repeated on a well‐established V‐Net to see if conclusion observation in proposed SC‐DenseNet can be extended to other networks. Subgroup analysis of performance: ASD and DSC performance were compared between subgroups (i) T1/2 vs. T3/4, (ii) using T1w, T2w, and a combination of both using independent *t*‐test.	V‐Net behave similarly as SC‐DenseNet in comparisons between the plain sequences and ce‐T1w, suggesting their results might extend to other network architectures. ASD was more substantial in T3/4 patients (*p* < 0.05), but DSC	Statistical analysis results were not in full agreement with key conclusion with paired‐*t*‐test statistical significance (*p* < 0.0167) between both DSC and ASD of model trained different modalities. Single‐center without external validation. Results generated from a single data‐split that can subject to selection bias.
[33]	2022	20 vs. 20 ?	LeNet/ResNet/EfficientNet	ce‐T1w‐fs	Utilized several 2D networks with publicly available pre‐trained weights for end‐to‐end classification of input slice into NPC and non‐NPC predictions.	One of the few studies that conducted repeated K‐fold cross‐validation which can minimize risks of selection bias through testing the performance with many different train‐test splits configurations.	Interpretability of trained networks: Qualitatively investigated Grad‐CAM to explore reliability of models. Value of pre‐trained weights: Compared performance of models that are trained from scratch and those that are trained with pre‐trained weights (publicly available weights from training on large volume of natural image classification tasks).	Compared to LeNet, other network structures with a deeper structure gave more relevant activation maps. Pre‐trained networks showed better performance than those trained from scratch.	Limited sample size. Single‐center without external validation. Patient inclusion/exclusion criteria was vague. Clinical information, such as T‐stage distribution, was not clearly reported. These factors led to “medium” risk of bias for this article.
[34]	2022	320 vs. 335 320/0/0/0	BB‐RENT + Radiomics	T2w‐fs	Made use of bagging, boosting and repeated elastic net techniques for stable radiomic feature selection to construct an ensemble signature for the discrimination of segmented lesion into NPC and non‐NPC.	Proposed methodology for improving stability of radiomic features selection.	Stability of radiomic features selection: Statistically compared stability of proposed features selection method with existing methods to verify improvements based on independent *t*‐test.	BB‐RENT showed significantly higher average Jaccard and Nogueira Score (both *p* < 0.001) for measuring feature overlap.	Single‐center without external validation
[35]	2024	300 vs. 314 300/0/0/0	Radiomics	T2w‐fs + ce‐T1w	Typical radiomics pipeline that extracts features from lesion on various MRI sequences, and built binary classification model to classify between those that belong to NPC and non‐NPC lesion	Multi‐centered study with external validation. One of the few studies to have performed reader study to verify accuracy changes in radiologists with and without aid by radiomics model.	Performance compared to radiologist: Compared performance of radiomics model with 3 radiologists using DeLong test. Radiologists aided by radiomics model:Compared the performance of the 3 radiologists with and without radiomics results. Compared performance of ce‐MRI with non‐contrast MRI: Compared performance of radiomics models built from plain scan only, and that built from ce‐MRI. Robustness test: Used DeLong test to compare ROC curves of the model on internal and external validation set.	AI performed better (best AUC 0.932) than all three radiologists (AUC = 0.707 to 0.818, all *p* < 0.05) for external validation set. Radiologists performance improved when aided by model with significant improvements (AUC = 0.782 to 0.873, all *p* < 0.05), performance of ce‐T1w only model and ce‐T1w + T2w‐fs performed significantly better than T2w‐fs only model (both p < 0.05). No significant difference between ce‐T1w only model and ce‐T1w + T2w‐fs model (*p* = 0.511). No significant differences in AUC of three radiomics models in the internal and external validation set performance (*p* = 0.073–0.389 & 0.140–0.505, respectively) [Note: Unclear how two *p* value ranges were resulted from DeLong test comparisons of internal and external validation performance in 3 models].	Results generated from a single data‐split that can subject to selection bias. Notable discrepancy of reader performance from other reviewed literature.

*Note:* * the character ‘/’ indicates methods or sequences were used in parallel, and the character ‘+’ indicates they were used in combination.

### 3.3 Risk of Bias and Applicability Concern Evaluation

#### 3.3.1 Risk of Bias

Based on the four categories QUADAS‐2 evaluation of the 38 included studies, 11 studies [16, 32, 34, 35, 36, 37, 38, 39, 40, 41, 42], 21 [14, 31, 33, 43, 44, 45, 46, 47, 48, 49, 50, 51, 52, 53, 54, 55, 56, 57, 58, 59, 60] and 6 studies [61, 62, 63, 64, 65, 66] were rated as having low, medium, and high risk of bias. Source of potential bias risk stemmed primarily from the omission of clinical details in their reports, including patients' clinical information, selection criteria, and the criteria to the formation of the reference standard (ground‐truth), corresponding to the “Patient selection” and “Reference Standard” domain of QUADAS‐2, respectively.

#### 3.3.2 Applicability Concerns

Among the 38 reviewed AI studies, 7 [14, 16, 31, 32, 33, 34, 35], 20 [36, 38, 39, 40, 41, 42, 46, 47, 51, 53, 54, 56, 57, 58, 59, 60, 63, 64, 65, 66], and 11 [37, 43, 44, 45, 48, 49, 50, 52, 55, 61, 62] studies were rated with low, high, and unclear applicability concerns for MRI screening of NPC, respectively. Once again, under‐reporting patients' clinical information and image acquisition protocol details resulted in “Unclear” judgment. In particular, T‐stage distributions of the included patients that are required to verify item 1 and/or 3 in Table 1 were unavailable from a large portion (21 of 38) of reviewed studies. Additionally, the MRI protocols utilized to acquire AI's input were unclear in roughly a quarter of the studies (9 in 38), from which the use of intravenous contrast‐enhanced MRI was uncertain for evaluation of item 4 in Table 1.

The inclusion of non‐NPC participants in the study cohort is another important factor for applicability concerns as they are the anticipated majority in screening service. Only 7 studies performing the discrimination task [14, 16, 31, 32, 33, 34, 35] have included non‐NPC controls; the vast majority of detection and localization studies did not evaluate AI performance in non‐cancer subjects. They have either omitted or only simulated non‐NPC samples with slices that did not capture the primary NPC in the field‐of‐view. Applicability concern and risk of bias evaluation results are tabulated in Table 4.

**TABLE 4:** Detailed evaluation of the risk of bias and applicability to Nasopharyngeal carcinoma (NPC) screening of included and reviewed artificial‐intelligence‐driven studies.

Study	Risk of bias					Applicability Concerns					
	P	I	R	FT	Overall	T1	N	D	PL	S	Overall
Z Ma, 2018	✓	✓	✗	✓	Med	?	✗	?	✓	✓	Unclear
Q Li, 2018	✓	?	✓	✓	Med	?	✗	?	✗	✓	Unclear
L Lin, 2019	✓	✓	✓	✓	Low	✓	✗	✗	✗	✗	High
F Guo, 2020	✓	?	?	✗	Med	?	✗	?	?	✓	Unclear
Y Fei, 2020	✓	?	?	✓	Med	?	✗	?	?	✓	Unclear
L Ke, 2020	✓	?	✓	✓	Med	✓	✓	✗	✗	✓	Low
Y Ye, 2020	✓	✓	✓	✓	Low	?	✗	?	?	✗	Unclear
H Wang, 2020	✓	✓	?	?	Med	?	✗	?	✗	✗	High
H Chen, 2020	✓	✓	?	?	Med	?	✗	?	✗	✗	High
D Wang, 2021	✗	?	✗	✓	High	?	✗	?	✗	✗	High
LM Wong [38], 2021	✓	✓	✓	✓	Low	✓	✗	✗	✓	✓	High
LM Wong [16], 2021	✓	✓	✓	✓	Low	✓	✓	✗	✓	✓	Low
P Tang, 2021	✓	✗	?	✗	High	?	✗	?	?	✓	Unclear
Y Li, 2021	✓	✓	?	✗	Med	?	✗	?	?	✓	Unclear
J Zhang, 2022	✓	?	?	✗	Med	?	✗	?	✗	✓	Unclear
R Huang, 2022	✗	✗	?	✗	High	✓	✗	✓	✗	✗	High
H Zhou, 2022	✓	?	✓	?	Med	?	?	?	?	✗	Unclear
L Ji, 2022	✓	?	✓	?	Med	?	✓	?	✗	✓	Low
Y Liu, 2022	✓	?	✗	✗	High	?	✗	?	✗	✓	High
LM Wong, 2022	✓	✓	✓	✓	Low	✓	✓	✗	✓	✓	Low
G Tao, 2022	✓	✓	✓	✓	Low	✓	✗	✗	✗	✗	High
Y Deng, 2022	✓	✓	✓	✓	Low	✓	✓	✗	✓	✓	Low
W Liao, 2022	✓	✓	✓	?	Med	✓	✗	✗	?	✓	High
Y Li, 2022	✓	✓	✓	?	Med	?	✗	?	✗	✗	High
S Li, 2022	✓	✓	✓	?	Med	?	✓	?	✓	✓	Low
X Luo, 2023	✓	✓	✓	✓	Low	✓	✗	✗	✗	✓	High
H Wu, 2023	✓	✓	?	?	Med	?	✗	?	?	✓	Unclear
Y Zeng, 2023	✓	✗	?	?	High	?	✗	?	?	✓	Unclear
K Yang, 2023	?	✗	✓	✓	Low	✓	✗	✗	✗	✓	High
Y Zhang, 2024	✓	?	✓	✓	Med	✓	✗	✗	✓	✓	High
Y Li, 2024	✓	✗	✓	✓	Med	?	✗	?	✗	✗	High
X Han, 2024	✓	✓	✓	✓	Low	✗	✗	✗	✗	✗	High
J Zhang, 2024	✓	✓	?	?	Med	?	✗	?	✗	✓	High
J Cheng, 2024	✓	✓	✓	✓	Low	✓	✓	✗	✗	✗	Low
Z Cai, 2024	?	✗	✗	✓	High	✓	✗	✗	✓	✗	High
R Mao, 2024	✓	✓	✗	?	Med	?	✗	?	✗	✓	Unclear
Y Qi, 2025	✗	✗	✓	?	Med	✓	✗	✗	✗	✗	High
X Luo, 2025	?	✓	✓	✓	Med	✓	✗	✗	✗	✓	High

*Note:* ✓ indicates low risk of bias or applicability concern; ✗ indicates high risk of bias or applicability concern; ? indicates unclear.

Abbreviations: D, NPC screening distribution; FT, flow and timing; I, index test; *N*, included non‐NPC participants; *P*, patient selection; PL, Non‐contrast MRI scan; *R*, reference standard; S, single sequence; T1, Included stage T1 NPC.

### 3.4 Meta‐Analysis of AIs' Performances

Out of 38 reviewed papers, 3, 32, and 7 studies devised or investigated AI for ROI detection, lesion segmentation, and discrimination, respectively, with 5 studies investigating multiple tasks; one study proposed to first detect an ROI bounding box then performed segmentation of primary NPC within it [39]; another one detected the ROI bounding box then performed discrimination of the content within [31]; the other two were from the same research group who proposed a self‐constrained AI that simultaneously performed both segmentation and discrimination [14, 32], all of these studies characterized lesions detected/segmented into either NPC or non‐NPC entities. The last multiple‐tasked study segmented and predicted the T‐stage of the primary NPC; the latter part was omitted and did not count towards any of the task roles as it is irrelevant to screening [42]. All reviewed studies utilized deep learning but three [34, 35, 49], which investigated radiomics with conventional machine learning.

For ROI detection, meta‐analysis was not practical in view of the heterogeneity of their implementation as well as the small number of only 3 studies [31, 39, 52] in this group. For segmentation of primary NPC which was most extensively studied among the three including 32 studies [14, 32, 36, 37, 38, 39, 40, 41, 42, 43, 44, 45, 46, 47, 48, 49, 51, 52, 53, 54, 55, 56, 57, 58, 59, 60, 61, 62, 63, 64, 65, 66], DSC was the primary metric for measuring performance reported in all studies, followed by ASD reported in 13 of 32 studies. Excluding two studies that only reported the combined performance of both primary NPC and nodal metastasis [37, 66], and two more where confidence intervals were unavailable [58, 66]. The pooled DSC and ASD were 0.80 (CI: 0.76–0.83) and 1.56 mm (CI: 1.11–2.00 mm), respectively. Detailed sensitivity analysis results are given in the Figure S1 and no studies were excluded given a nice overlap of confidence intervals.

For discrimination, 7 studies [14, 16, 31, 32, 33, 34, 35] reported discriminating NPC with non‐NPCs including benign hyperplasia or chronic inflammation MRI [14, 16, 31, 32, 34], normal NP [33], and also other head and neck diseases [31]. One study reported simultaneous segmentation and T‐stage classification and was not further considered as it is a post‐diagnosis step [42]. All 7 studies modeled the problem as binary classifications of the input into either NPC or non‐NPC predictions. Area under the curve (AUC) and sensitivity were the primary metrics reported for discrimination, with all studies utilizing deep learning, except two that utilized radiomics with conventional machine learning [34, 35]. Excluding one study that did not report the sensitivity and specificity of their models but only a range of AUCs [31], the pooled AUC was 0.96 based on SROC and the pooled sensitivity and specificity were 97.1% (CI: 88.0%–99.3%) and 87.8% (CI: 78.9%–93.2%), respectively. Two pairs involving four studies showed significant risk of cohort overlap ([15, 34]; [14, 32]), but sensitivity analysis showed minimal impact of only ±2% to pooled sensitivity (95.3%–97.5%) and specificity (85.2%–88.5%). Detailed sensitivity analysis is given in Figure S2, Table S3 and no studies were excluded based on that.

**FIGURE 4:**
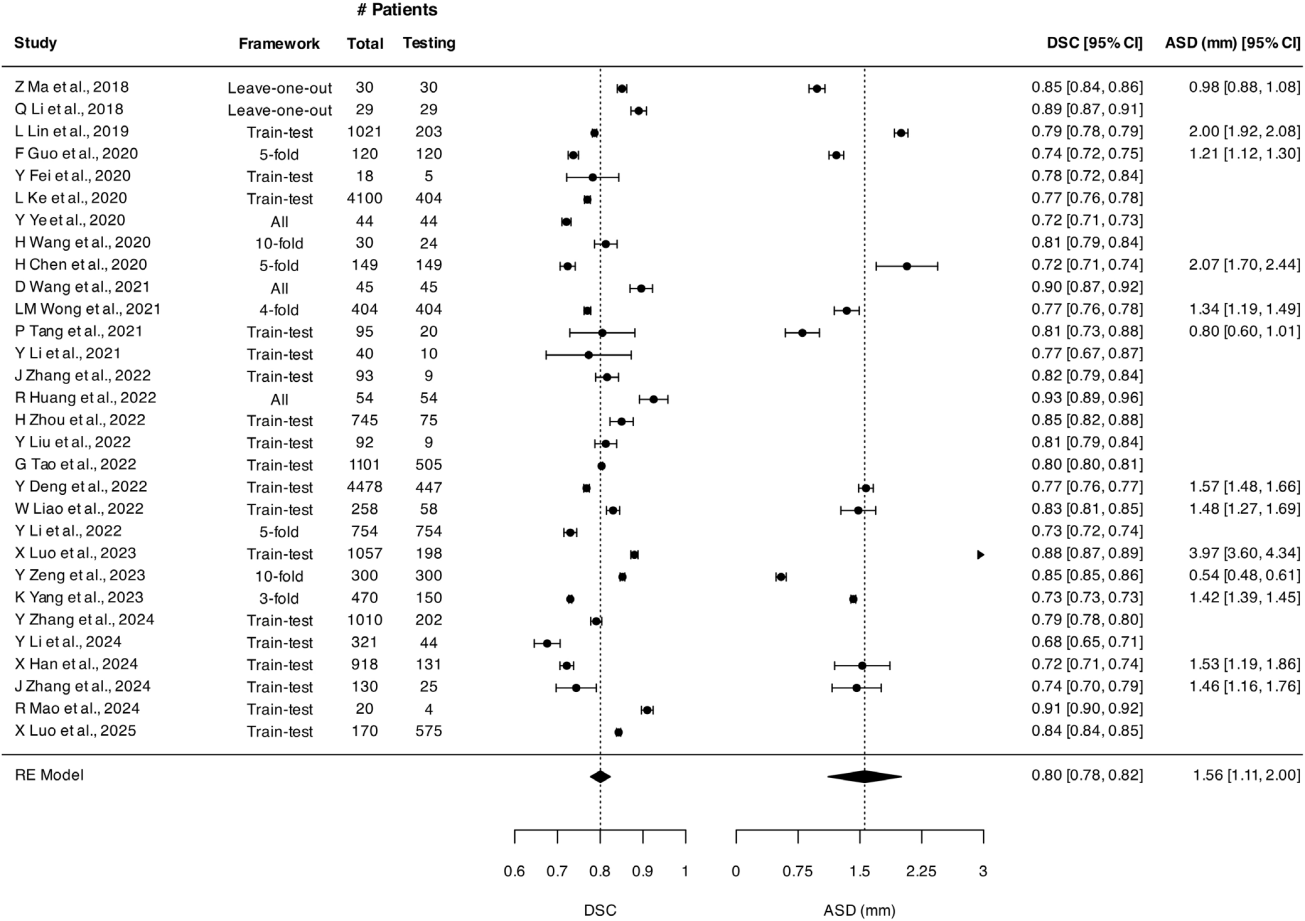
Forest plot of AI performance for segmentation of NPC on MRI in Dice similarity coefficient (DSC) and average surface distance (ASD). Dotted line represents the random effect model estimation of pooled performance. For validation framework, studies with “All” labels, representing the utilization of all patients for both training and testing, should be interpreted with caution as this practice is generally considered to generate inflated performance. In view of sensitivity analysis showed minimal effects on pooled performance, they were retained in this forest plot. CI = confidence interval, RE = random effect.

**FIGURE 5:**
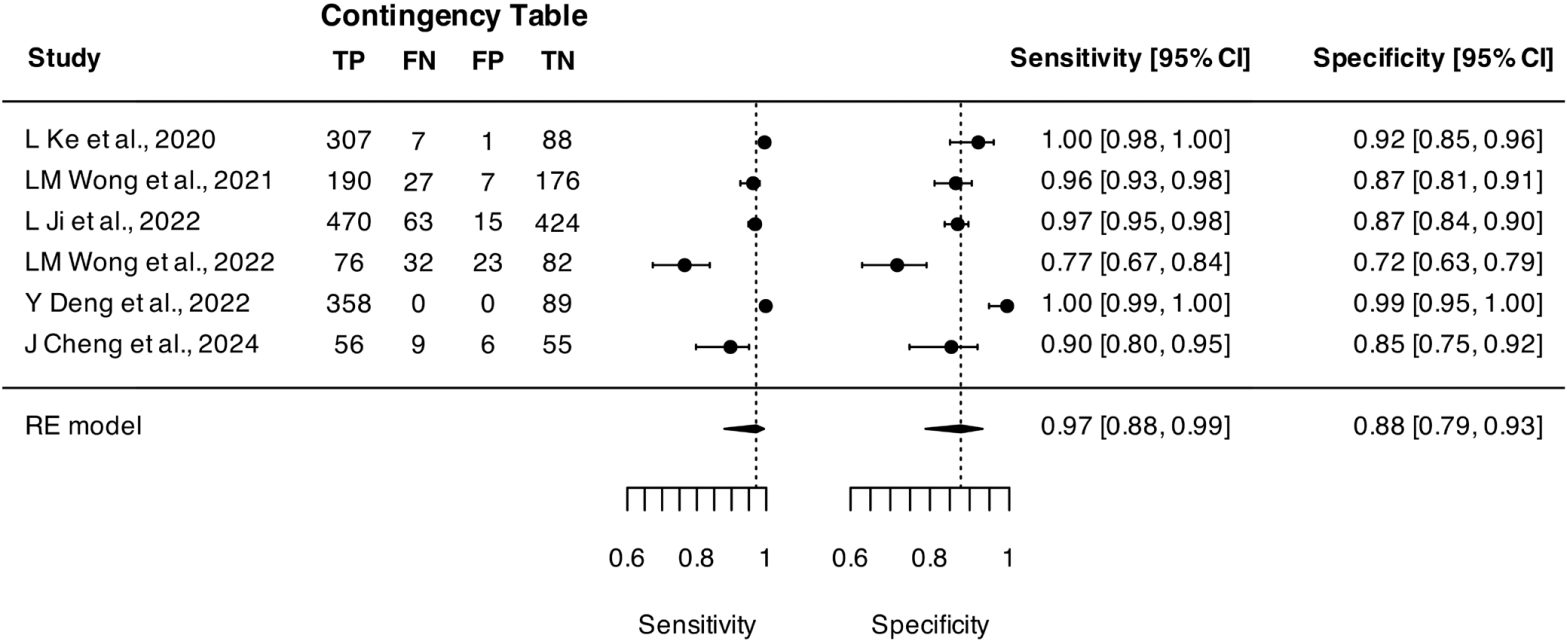
Forest plot of AI performance for discrimination of NPC and non‐NPC on MRI in sensitivity and specificity. Noted that one discrimination study did not report the sensitivity and specificity of their method was also excluded. CI = confidence interval, TP = true‐positive, TN = true‐negative, FP = false‐positive, FN = false‐negative, RE = random effect.

Results are summarized in Table 5, and in forest plot Figure 4 and Figure 5.

**TABLE 5:** Summary table of performance of reviewed studies.

References	Year	MRI sequence	Included patients	T‐stage distribution (T1/T2/T3/T4 [; Non‐NPC])	Patient split^a^ (Train [/Validation]/Test)	Segmentation (Mean ± SD)		Discrimination			Detection
						ASD (mm)	DSC	Sens	Spec	AUC	IoU
[4]	2018	T1w	30	?	Leave‐one‐out	0.98 ± 0.27	0.85 ± 0.03	—	—	—	—
[50]	2018	DCE	29	?	No split	—	0.89 ± 0.05	—	—	—	—
[36]	2019	T1w + T2w + ce‐T1w + ce‐T1w‐fs	1021	50/123/601/247	818/203	2.00 ± 0.60	0.79 ± 0.04	—	—	—	—
[44]	2020	ce‐T1w	120	?	5‐fold CV	1.21 ± 0.50	0.74 ± 0.07	—	—	—	—
[49]	2020	ce‐T1w‐fs	18	?	13 (10‐fold CV)/5	—	0.78 ± 0.07	—	—	—	—
[14]	2020	ce‐T1w	4100	739/297/1547/559; 958	3285/411/404	—	0.77 ± 0.07	99.70%	91.70%	0.96	—
[37]	2020	T1w + T2w	44	?	10‐fold CV	—	0.72 ± 0.04	—	—	—	—
[56]	2020	T1w + T2w + ce‐T1w	24	?	10‐fold CV	—	0.81 ± 0.07	—	—	—	—
[57]	2020	T1w + T2w + ce‐T1w	149	?	5‐fold CV	2.07 ± 2.32	0.72 ± 0.11	—	—	—	—
[65]	2021	T1w + T2w‐fs + ce‐T1w	45	?	No split	—	0.90 ± 0.09	—	—	—	—
[38]	2021	T2w‐fs	404	130/59/140/75	4‐fold CV	1.34 ± 1.55	0.77 ± 0.08	—	—	—	—
[61]	2021	ce‐T1w‐fs	95	?	60/15/20	0.80 ± 0.47	0.81 ± 0.17	—	—	—	—
[55]	2021	ce‐T1w‐fs	40	?	30/10	—	0.77 ± 0.16	—	—	—	—
[16]	2021	T2w‐fs	412	203/0/0/0; 202	3‐fold CV	—	—	92.40%	90.60%	0.96	—
[33]	2022	ce‐T1w	40	?	?‐fold CV	—	—	97.00%	87.10%	0.98	—
[34]	2022	T2w‐fs	655	320/0/0/0; 335	442 (5‐fold CV)/213	—	—	76.80%	71.90%	0.80	—
[45]	2022	ce‐T1w	93	?	~74/10/9	—	0.82 ± 0.04	—	—	—	—
[31]	2022	T2w	2485	?	1866/619	—	—	?	?	0.97^b^	—
[64]	2022	ADC + DCE + T2w‐fs	54	I: 18/II: 22/III: 14	No split	—	0.93 ± 0.12	—	—	—	—
[48]	2022	T1w + T2w	745	?	670/75	—	0.85 ± 0.12	—	—	—	—
[63]	2022	ce‐T1w‐fs	92	?	~74/9/9	—	0.81 ± 0.04	—	—	—	—
[39]	2022	T1w + T2w + ce‐T1w	1101	?	301/129/166 + Ext: 505	—	0.80 ± 0.03	—	—	—	69.7%
[32]	2022	T1w/T2w/ce‐T1w	4478	802/364/1776/640;896	3582/448/448	1.57 ± 0.95	0.77 ± 0.07	99.70%	100.00%	1.00	—
[53]	2022	T1w/T2w/ce‐T1w‐fs	258	T1 + T2: 101/T3 + T4: 157	180/20/58	1.48 ± 0.82	0.83 ± 0.06	—	—	—	—
[54]	2022	T1w + T2w + ce‐T1w	754	?	5‐fold CV	—	0.73 ± 0.21	—	—	—	—
[41]	2023	ce‐T1w	1057	114/229/504/210	600/259/198	3.97 ± 2.69	0.88 ± 0.06	—	—	—	—
[62]	2023	T1w	300	?	10‐fold CV	0.54 ± 0.55	0.85 ± 0.05	—	—	—	—
[52]	2023	T2w	800	?	640/160	—	—	—	—	—	79.0%
[35]	2024	ce‐T1w + T2w‐fs	614	300/0/0/0; 314	390/98/Ext: 126	—	—	90.30%	85.90%	0.93	—
[46]	2024	T1w	1010	507/503/0/0	606/202/202	—	0.79 ± 0.09	—	—	—	—
[47]	2024	T1w + T2w + ce‐T1w‐fs	321	?	249/28 + Ext: 44	—	0.68 ± 0.10	—	—	—	—
[40]	2024	T2w + ce‐T1w‐fs	918	62/103/454/284	451/151/151 + Ext: 165*	1.53 ± 1.96	0.72 ± 0.09	—	—	—	—
[51]	2024	ce‐T1w‐fs	130	?	90/15/25	1.46 ± 0.76	0.74 ± 0.12	—	—	—	—
[42]	2024	ce‐T1w	620	26/140/87/67^c^	470 (3‐fold) + Ext: 150	1.42 ± 0.19	0.73 ± 0.02	—	—	—	—
[58]	2025	T1w + T2w + ce‐T1w	210	40/70/84/16	155 (5‐fold) + Ext: 55	—	Excluded^d^	—	—	—	—
[59]	2025	ce‐T1w	1227	144/269/572/242	Atypical	—	0.84 ± 0.06	—	—	—	—
[66]	2025	T1w + T2w	116	?	Atypical	—	Excluded^e^	—	—	—	—
[60]	2025	T1w	20	?	16/4	—	0.91 ± 0.01	—	—	—	—

*Note:* Rows are ordered chronologically according to publication year. * the symbol “/” indicates that models or sequences were tested in parallel, whereas “ + ” indicates that the methods were used in an inseparable combination.

Abbreviations: ? = unclear; ADC = apparent diffusion coefficient map; ASD = average surface distance; ce‐T1w = contrast‐enhanced T1‐weighted; CV = cross‐validation; DCE = dynamic contrast‐enhanced scan; DSC = Dice similarity score; IoU = intersection over union; T2w‐fs = T2‐weighted fat‐suppressed.

^a^ “No split” means study did not split the included data and used the same data for training and validation, “Ext” means data was from an external center that did not contribute to the training set, “~” symbol indicate the article have not provided the exact number but rather just mentioned the ratio, “*” indicates potential misalignment of numbers stated.

^b^ Value is the best average AUC across multiple binary classifications that predicts 5 different head and neck diseases reported by the study.

^c^ External data demographics not stated.

^d^ Excluded from meta‐analysis because no uncertainty metrics provided.

^e^ Excluded from meta‐analysis because performance were calculated without separating primary and node segmentation.

### 3.5 Subgroup Analysis for Factors Impacting Performance

Meta‐regression was performed for segmentation and discrimination tasks to understand if the NPC screening applicability requirements of the input, namely (a) not requiring ce‐MRI, and (b) using only 1 sequence are significant factors that affected the performance. Stage distribution and inclusion of stage T1 patients could not be investigated because too many studies (24 in 38) did not report T‐stage distribution details.

For the use of contrast‐enhanced scans (a), it did not demonstrate a significant impact on performance for either segmentation or discrimination tasks. No statistical significance was observed for segmentation in terms of DSC (*p* = 0.811) or ASD (*p* = 0.182) in segmentation, with the pooled performance of AI models using non‐contrast MRI scans (*n* = 4) and contrast‐enhanced scans (*n* = 18) being 0.80 (CI: 0.76–0.83) and 0.80 (CI: 0.77–0.84), respectively, for DSC, and 1.30 mm (CI: 0.95–1.64 mm) and 2.07 mm (CI: 1.31–2.84 mm), respectively, for ASD. Eight studies were excluded from this analysis due to unclear MRI input details for their proposed methods. Similarly, no statistical significance was observed for discrimination for neither sensitivity nor specificity, both with and without exclusion of radiomics studies (*p* = 0.73 & 0.65 and *p* = 0.99 & 0.97, respectively) corresponding to use of contrast‐enhanced scans. The pooled results for non‐contrast MRI studies (*n* = 3) and contrast‐enhanced scans studies (*n* = 3) were close with major overlap in confidence intervals, at 97.5% (CI: 58.9%–99.9%)/89.9% (CI: 60.7%–98.1%) and 97.6% (CI: 85.7%–99.6%)/88.1% (CI: 82.2%–92.3%), respectively.

For the use of multiple MRI sequences (b), again no significant influence on AI performance for segmentation was observed, while its role in discrimination could not be fully assessed due to limited data. For segmentation, no statistical significance was observed for the “single sequence” versus “multiple sequences” moderator in terms of DSC (*p* = 0.318) or ASD (*p* = 0.470). The pooled performance of AI models using a single sequence (*n* = 19) and multiple sequences (*n* = 11) was 0.79 (CI: 0.74–0.83) and 0.81 (CI: 0.79–0.84), respectively, for DSC, and 1.88 mm (CI: 1.57–2.19 mm) and 1.47 mm (CI: 0.90–2.04 mm), respectively, for ASD. For discrimination, subgroup analysis for the “multiple sequences” factor was not feasible, as all but one study employed a single sequence in their final model.

Additionally, while comparison between deep learning (DL) and non‐DL methods was not feasible for the segmentation task because only one study utilized non‐DL methods, discrimination tasks moderator analysis revealed that the DL methods (*n* = 4) are significantly better than radiomics (*n* = 2) as classifiers. A significant difference in pooled sensitivity (98.8% [CI: 94.7%–99.7%] vs. 84.3% [CI: 65.2%–93.9%]; *p* = 0.013) and a marginally insignificant effect on specificity (91.0% [CI: 84.1%–95.1%] vs. 79.4% [CI: 61.4%–90.4%]; *p* = 0.07) were observed for DL vs. non‐DL studies.

**FIGURE 6:**
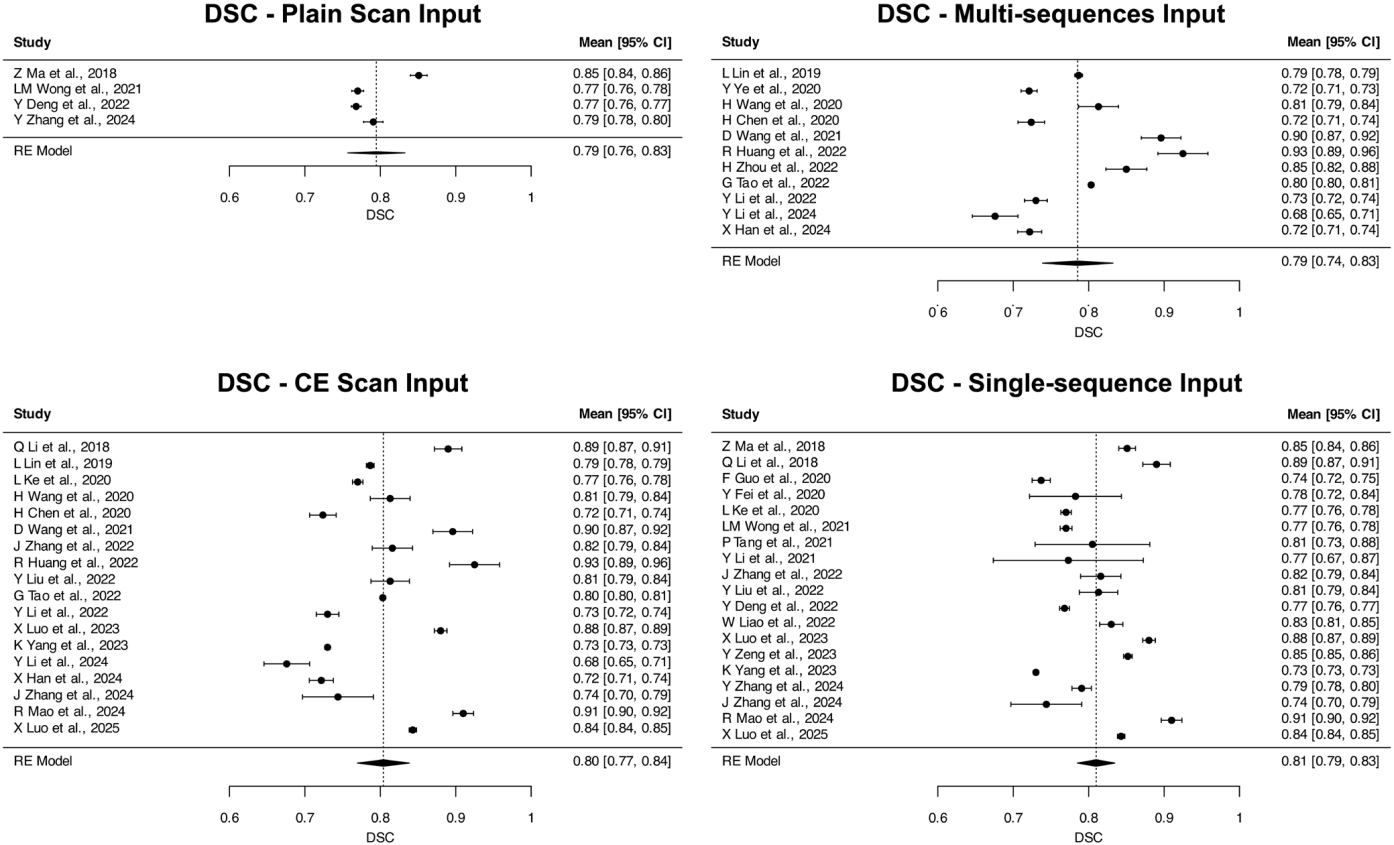
Subgroup analysis of AIs' segmentation performance measured by Dice similarity coefficients (DSC). Studies were grouped according to two dichotomous MRI protocol factors—whether proposed AI operates on plain‐scan and utilize only one single sequence—for subgraph meta‐analysis. Studies that did not clearly reported the status of these factors were excluded from both the full analysis and the subgroup analysis. CI = confidence interval, RE = random effect.

**FIGURE 7:**
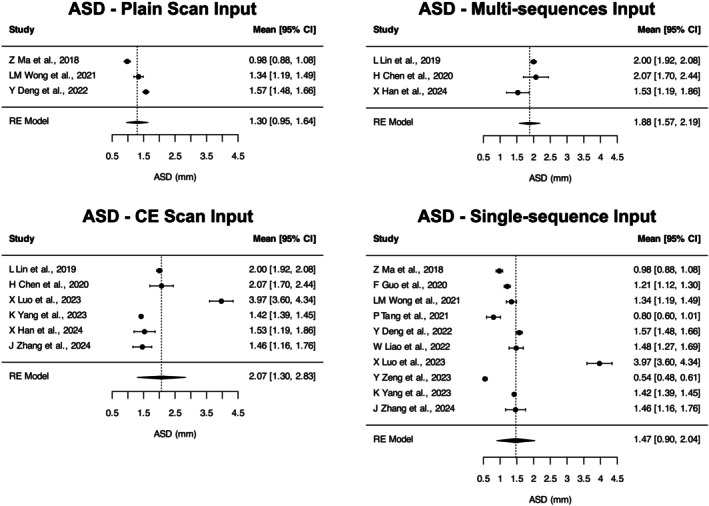
Subgroup analysis of AIs' segmentation performance measured by Average Surface Distance (ASD). Studies were grouped according to two dichotomous MRI protocol factors—whether proposed AI operates on plain‐scan and utilize only one single sequence—for subgraph meta‐analysis. Studies that did not clearly reported the status of these factors were excluded from both the full analysis and the subgroup analysis. CI = confidence interval, RE = random effect.

**FIGURE 8:**
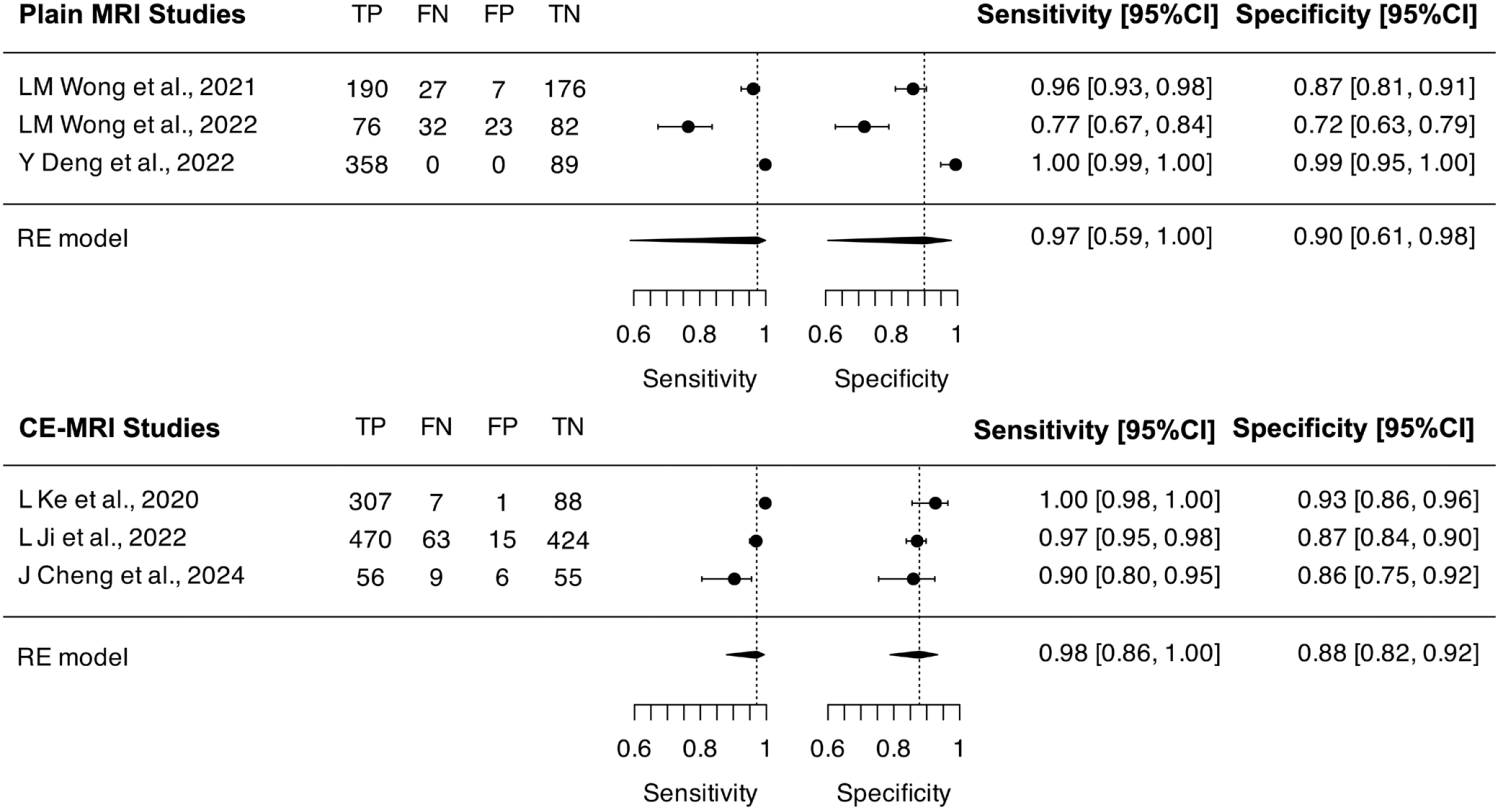
Subgroup analysis of AIs' characterization performance to discriminate existing lesion between NPC and non‐NPC entities, grouped by whether the proposed AI method require contrast‐enhanced (CE) scans. CI = confidence interval, RE = random effect.

**FIGURE 9:**
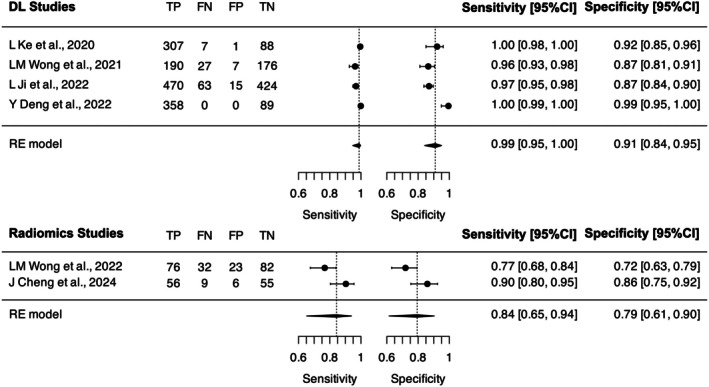
Subgroup analysis of AIs' characterization performance to discriminate existing lesion between NPC and non‐NPC entities, grouped by whether the AI was developed based on deep learning (DL) or conventional machine learning with radiomics. CI = confidence interval, RE = random effect.

Detailed forest plots for these subgroup analyses of segmentation (Figures 6, 7) and discrimination studies (Figures 8, 9) are provided.

### 3.6 Heterogeneity Analysis

For both DSC and ASD, the heterogeneity of the pooled studies was high at $I^{2}$ = 99.5% and 99.6%, respectively. The Q statistics were 3869.36 (*p* < 0.05) and 1234.77 (*p* < 0.05) for DSC and ASD, respectively. Nevertheless, the magnitude of $\tau^{2}$ was small at 0.004 and moderate at 0.659 when compared to the pooled value (0.80 and 1.56 mm) for DSC and ASD, respectively. In contrast, the heterogeneity of discrimination studies covering both sensitivity and specificity was small at $I^{2}$ = 1.5%−12.3%.

## 4 Discussion

This review included 38 NPC detection, segmentation, and discrimination studies in 23,398 patients in which the proposed MRI AI algorithms may potentially be applied for MRI‐based NPC screening. Contrary to existing literature reviews and meta‐analysis [18, 19, 20, 21], we specifically reviewed these AI studies under the context of NPC screening with MRI, which has unique concerns compared to regular diagnosis and management. Specifically, we found AI algorithms demonstrated good performance in lesion segmentation as well as in lesion discrimination. Furthermore, our meta‐analysis revealed that the use of intra‐venous contrast‐enhanced MRI, which usage is limited in screening, showed no statistically significant performance differences for both segmentation (*p* > 0.05 for both DSC and ASD) and discrimination task (*p* > 0.05 for both sensitivity and specificity) compared to non‐contrast MRI inputs. These findings suggest the promising potential of AI to enhance NPC screening workflows.

### 4.1 Answering the Raised Research Questions

With respect to our first query, results shown by segmentation and discrimination studies indicate AI showed promise for screening NPC with MRI, but we were unable to systematically evaluate ROI detection algorithms due to the limited number of studies. For NPC segmentation, when examining the pooled results of AI compared to those of humans, we find a comparable to better performance by AI. AI achieved a DSC of 0.80 (CI: 0.78–0.82) for segmentation pooled across 31 studies. This pooled performance lies at the upper range of the inter‐ and intra‐observer variability among clinicians and expert researchers reported in the literature for segmentation of primary NPC. Three studies reported variability ranging from a DSC of 0.70 to 0.80 [36, 38, 58], while another documented a substantially lower DSC of 0.642 between two medical experts [44]. This indicates AI has achieved a segmentation accuracy comparable to or better than the agreement achieved among human experts.

For discrimination AI, they showed a promising sensitivity/specificity of 97.1% (CI: 88.0%–99.3%)/87.8% (CI: 78.9%–93.2%) pooled across 6 studies. This performance is comparable to those reported in previous meta‐analyses of radiologists' performance (evaluating all MRI sequences) at 97.1% (CI: 95.2%–99.3%)/91.7% (CI: 88.3%–94.2%) [67]; and comparable to better than a recent prospective radiologist reader study that focused on only non‐contrast‐enhanced MRI at 88.9%/91.1% in asymptomatic patients who underwent EBV‐DNA NPC screening with positive results [13]. Compared to the standard‐of‐care diagnosis pathway, endoscopy, AI displayed a better sensitivity and comparable to better specificity than those reported in meta‐analyses for white‐light endoscopy 75.9% (CI: 64.9%–84.3%)/83.9% (CI: 51.3%–96.3%) and narrow‐band imaging endoscopy 87.1% (CI: 80.8%–91.5%)/90.5% (CI: 81.6%–95.3%) [68].

Furthermore, answering our second query regarding studied imaging protocols, meta‐analysis revealed no significant performance differences between AI models using non‐contrast versus contrast‐enhanced MRI for both segmentation (*p* > 0.05 for both DSC and ASD) and discrimination between NPC and non‐NPC entities (*p* > 0.05 for both sensitivity and specificity). This suggests AI models have the capability to extract sufficient information from non‐contrast MRI alone in performing the task of interest, indicating the possibility of exempting intravenous contrast administration without having to sacrifice performance in NPC screening scenarios. Nevertheless, the pooled CI of both sensitivity and specificity for non‐contrast MRI discrimination studies showed a notably larger range (Sensitivity/Specificity CI: 58.9%–99.9%/60.7%–98.1%) than those pooled from contrast‐enhanced scan studies (CI: 85.7%–99.6%/82.2%–92.3%), which suggests a latent source of variability for discrimination tasks that was not observed for segmentation tasks. This calls for further investigation to understand the underlying cause of such variability. Therefore, even though our pooled results showed no significant differences, these estimations are yet to be fully validated for incorporating into screening protocols. For the moderator analysis of multi‐sequence versus single‐sequence input protocols, though segmentation AI showed no significant influence (*p* > 0.05), its effect on discrimination AI could not be assessed as only 1 in 7 reviewed studies utilized multiple sequences. Similarly, the limited number of non‐DL AI studies for segmentation precluded statistical analysis of DL versus non‐DL. Though DL intuitively outperformed non‐DL for discrimination (*p* < 0.05), the large ratio of sample size (*n* = 4 & 2 for DL & radiomics, respectively) rendered the comparison less conclusive. In summary, we found intravenous contrast‐enhancement can potentially be exempted without sacrificing performance for DL algorithms.

Nevertheless, with respect to our final query, reviewed studies showed significant applicability pitfalls, with only 7 discrimination studies having a low applicability concern. High and unclear applicability concerns were primarily associated with the under‐representation of non‐NPC patients in the tested cohort. All studies except these 7 discrimination studies either did not incorporate any non‐NPC patients or under‐reported the patient demographics of their dataset such that the distribution is unclear. While this design is arguably logical for studies focusing on NPC segmentation, it also restricts the developed AI's applicability to patients with confirmed NPC only. Consequently, the performance of NP lesion localization through ROI detection or segmentation AI in the presence of benign hyperplasia of NP is a knowledge gap that requires further studies to address. Moreover, the under‐reporting of the patient demographics also raises the risk‐of‐bias concerns. Crucial clinical information was under‐reported in many of the included articles, including NPC T‐stage (21 out of 38 studies) and MRI protocols (9 out of 38 studies). These factors can also significantly impact the reliability of reported AI performance as early stage disease is more challenging to detect. For instance, an arbitrary inclusion of more T3–T4 stage patients might lead to overstatement of performance, where improved performance could simply reflect the ease of detection rather than a superior AI algorithm. Overall, the existing AIs are not directly applicable to screening, but the bottleneck is primarily the lack of studies with appropriate validation strategies rather than technical constraints.

### 4.2 Outlook for Translation for AI‐Driven NPC Screening With MRI

Building on these queries, integration of AI‐driven MRI evaluation into clinical workflows is a promising avenue to address existing challenges in NPC screening. Currently, EBV‐related serology, including plasma DNA and antigen testing, is the primary method for screening NPC, but its low positive predictive value (PPV), ranging from 4.3% to 10.0% in previous prospective studies [4, 5, 6, 7] limits its standalone utility. Plain‐scan MRI and endoscopy are both viable downstream diagnostic modalities that offer higher PPVs of 34.8% and 35.0%, respectively [13], which are higher than those for EBV‐screen‐positive patients and so complement serology for test‐positive patients. Our findings suggest that AI‐driven plain‐scan MRI workflows could further enhance the role of MRI by reducing reliance on intra‐venous contrast agents and potentially simplifying the process compared to endoscopy, which often requires specialist involvement with limited room for implementing AI assistance. By establishing the feasibility of AI applications in plain‐scan MRI, this study highlights the potential for AI to augment existing screening strategies and improve accessibility, particularly in settings with limited access to experienced specialists.

## 5 Limitations

Our study has some limitations. First, AI segmentation performance exhibited significant heterogeneity, as indicated by a high Q statistic, $I^{2}$ values for both DSC and ASD measurements, affecting ASD more than DSC based on the $\tau^{2}$ (0.004 and 0.722, respectively). This high heterogeneity is consistent with previous reports [20], and likely stems from both the highly configurable nature of AI model development and validation steps. There exist many configurable hyperparameters including learning rate, momentum, class weights, stopping criteria…etc., and because they are rarely fully reported and exhibit high variability across studies that do, they are challenging to be fully considered during meta‐analysis. The heterogeneity for localization studies was additionally compounded by the variable combinations of MRI sequences used across studies, as no consensus exists on the optimal non‐contrast sequence for NPC screening such that primary source of training AI seems to depend on the institutional NPC diagnosis or staging practice. More importantly, the validation steps of segmentation are more versatile. For instance, averaging of metrics can disjoint at different levels such as at a 2D‐slice or 3D‐volume level, and across multiple folds in k‐fold cross‐validation, but despite their importance they are not always stated. Additionally, manually segmented references were also susceptible to inter‐/intra‐observer variability. These factors might have further widened the observed heterogeneity for segmentation tasks. Regardless, the small $\tau^{2}$ = 0.004 compared to pooled DSC (0.80) indicated though differences did not arise from sampling error, the magnitude of differences are minimal. Furthermore, the heterogeneity was not as high for discrimination studies as for segmentation studies, and sensitivity analysis for segmentation did not show any of the studies have significantly shifted the pooled performance. Second, to avoid adding complexity, this study excluded MRI AI studies that explicitly stated the segmentation of primary NPC was for radiotherapy treatment planning. This decision was made to rule out impact of latent differences in the “ground‐truth” that may present, such as requirements for treatment planning might entail more inclusive segmentation than diagnosis, to ensure dosage delivery. Third, we were unable to pool the performance for ROI detection in NPC screening, not only because there were insufficient studies for meta‐analysis and systematic review, but also their diverse usage of performance metrics, unlike in the case of segmentation and discrimination.

## 6 Conclusions

In conclusion, AI has shown promise in individual tasks on MRI that would be important for screening NPC. The pooled DSC for segmentation was 0.80 (CI: 0.78–0.82) indicating good localization accuracy, and sensitivity and specificity for discriminating between NPC and non‐NPC at 97.1% (CI: 88.0%–99.3%) and 87.8% (CI: 78.9%–93.2%), respectively. Additionally, the use of contrast‐enhanced sequences did not translate into significant improvement in AI performance, implying possibility to perform non‐contrast scans alone for screening. But while we highlighted the potential of AI, such potential is yet to be fully affirmed as only a fraction of the studies showed low applicability concerns that limited the statistical power of meta‐analysis, knowledge gaps towards translation remain. Notably, the typical practice to omit non‐NPC patients in ROI detection and segmentation studies is a blind spot that can limit translation for scenario prior to disease confirmation. In the future, studies should also focus on conducting controlled comparisons, preferably in a prospective design that is currently absent, in diagnosis performance of readers with and without aid of AI results to verify their efficacies. Filling these knowledge gaps will lay the final stone for a practical and reliable AI‐driven NPC screening protocol.

## Funding

This study is supported by the University Grant Council of Hong Kong, General Research Fund (Ref. GRF 14104221).

## Conflicts of Interest

The authors declare no conflicts of interest.

## Supporting information


**Table S1:** Exact search clause used for literature search for each online database.
**Table S2:** CLAIM checklist items and their relevance with domains defined in QUADAS‐2. This labeled checklist was used as a reference for evaluating the risk of bias in the reviewed articles.
**Table S3:** Pairwise drop sensitivity analysis to investigate the potential influences of potential overlap in patient cohorts for discrimination studies. Sens = sensitivity, Spec = specificity, CI = confidence interval.
**Figure S1:** Leave‐one‐out sensitivity analysis of the pooled AI primary NPC segmentation performance in terms of (Top) Dice similarity score (DSC) and (Bottom) average surface distance (ASD). The forest plot shows the pooled performances, their confidence range, and the heterogeneity measures Q statistics, I2, and τ2 with the study labeled as row header excluded from the meta‐analysis. For DSC, similar pooled results across studies were observed. But for ASD, one study (X Luo et al. 2023) showed apparent deviation from the rest of the studies. The “Overall” row shows the pooling without exclusion of outlier study.
**Figure S2:** Leave‐one‐out sensitivity analysis of the pooled AI primary NPC discrimination performance. Radiomics studies (LM Wong 2022 & J Cheng 2024) were the main contributors of heterogeneity. Therefore, they were excluded from the analysis. Here we only showed the I2 because the bivariate meta‐regression was conducted in log‐transformed space, making the interpretation of Q statistics and τ2 difficult.
