# Supplementary material for: Systematic Review of AI‐Driven Applications for Screening Nasopharyngeal Carcinoma Using MRI
Source: J Magn Reson Imaging. 2026 Jul 26;64(3):593–615. doi: 10.1002/jmri.70445 (PMC13466594; doi:10.1002/jmri.70445)
Supplement: Supplementary file 1 [file JMRI-64-593-s001.docx]

# Details of Search Keywords

Table S1	Exact search clause used for literature search for each online databases.

| Database/Search Engine | Search Clause |
| --- | --- |
| Pubmed | (("MRI"[Title/Abstract] OR "magnetic resonance imaging"[Title/Abstract] OR "MR"[Title/Abstract]) AND ("nasopharyngeal carcinoma"[Title/Abstract] OR "nasopharyngeal cancer"[Title/Abstract]) AND ("screening"[Title/Abstract] OR "detection"[Title/Abstract] OR "segmentation"[Title/Abstract] OR "discrimination"[Title/Abstract]) AND ("radiomics"[Title/Abstract] OR "deep learning"[Title/Abstract] OR "machine learning"[Title/Abstract] OR "artificial intelligence"[Title/Abstract] OR "neural network"[Title/Abstract])) NOT ("review"[Title] OR "commentary"[Title] OR "letter to the editor"[Title]) |
| Scopus | TITLE-ABS-KEY((MRI OR "magnetic resonance imaging" OR MR) AND ("nasopharyngeal carcinoma" OR "nasopharyngeal cancer") AND (screening OR detection OR segmentation OR discrimination) AND (radiomics OR "deep learning" OR "machine learning" OR "artificial intelligence" OR "neural network")) AND NOT TITLE(review OR commentary OR "letter to the editor") |
| Embase (Ovid-based syntax) | (('mri' or 'magnetic resonance imaging' or 'MR') and ('nasopharyngeal carcinoma' or 'NPC' or 'nasopharyngeal cancer') and ('screening' or 'detection' or 'segmentation' or 'discrimination') and ('radiomics' or 'deep learning' or 'machine learning' or 'artificial intelligence' or 'neural network')).ab,hw,kf,kw,ot,ti not ('review' or 'commentary' or 'letter to the editor')).ti. |

# Details of study inclusion and eligibility criteria

## Inclusion criteria

To ensure the inclusion of studies closely aligned with the context of screening, the literature was evaluated based on the following inclusion criteria:

1. The study proposed and evaluated an AI-driven algorithm utilizing either radiomics with conventional machine learning or deep learning;
2. The algorithm was designed to detect and/or characterize lesions in the nasopharynx;
3. The study investigated MRI data of patients with nasopharyngeal carcinoma (NPC);
4. The study population was from endemic region(s) of NPC.

and studies were excluded to ensure relevance to MRI screening setting based on:

1. The AI-driven algorithm required additional imaging inputs beyond MRI (e.g., CT). However, additional clinical record inputs collected prior to treatment were accepted;
2. The study focused on post-diagnosis steps, such as treatment planning, outcome prediction, or survival analysis.

We only include studies from endemic region because there are the significant differences in pathology and demographics between endemic and non-endemic region populations, this review is restricted to articles that examined a patient population from endemic regions only. Furthermore, the criteria to exclude studies that focused on post-diagnosis steps aims to reduce bias due to differences in purpose of the labelling. For example, segmentation studies that mentioned their aim is to segment the clinical target volume will be excluded from this review as labelling for therapeutic

## Eligibility

This review is confined to peer-reviewed journal articles reporting original studies written in English. All other types of indexed documents such as books, conferences proceedings, letters to the editor, editorial comments, pre-prints and other literature reviews, were not eligible for inclusion.

# Details of using the CLAIM checklist for risk of bias evaluation

In line with the spirit of QUADAS-2 for risk-of-bias judgement, we tailored the signaling questions based on the Checklist for Artificial Intelligence in Medical Imaging (CLAIM) ^1^ to fit the purposes of this systematic review. Specifically, we first categorized items in CLAIM according to their relevance to the four domains recommended in QUADAS-2 (Table S2). Then, for each included study, the CLAIM checklist items that are not marked as “N/A” in the Table S2 were evaluated to either “0” or “1”, with “1” indicating it is absent from the reviewed study. For items evaluated as “0” (present in the study), the corresponding text sentences/paragraphs that associate with the evaluation were recorded in a separate table as suggested by CLAIM authors.

The “Reference standard” section of the CLAIM checklist was further customized to reflect the application categories of the included studies. Specifically, item 14 was revised to incorporate the validity of the reference standard. For localization studies, item 14 was considered fulfilled (scored “0”) only when ground-truth labeling involved radiologists or oncologists. For discrimination studies, item 14 was considered fulfilled (scored “0”) only when the reference standard for NPC included histological verification and the reference standard for non-NPC included follow-up of at least 1 year.

After all studies were reviewed using this system, two reviewers independently evaluate the risk of bias with reference to the results as well as the captured text sentences/paragraphs. The risk of bias for each of the four QUADAS-2 domains were judged as “Low”, “High” or “Unclear” with consideration of item listed Table S2. Finally, the overall risk of bias of each study was rated with a three-points scale into either “Low” “Medium” or “High”.

Table S2	CLAIM checklist items and its relevance with domains defined in QUADAS-2. This labelled checklist was used as a reference for evaluating the risk of bias in the reviewed articles.

| **Section / Topic** | **No.** | **Item** | **Domains in QUADAS-2** |
| --- | --- | --- | --- |
| ***TITLE / ABSTRACT*** |  |  |  |
|  | 1 | Identification as a study of AI methodology, specifying the category of technology used (e.g., deep learning) | N/A |
| ***ABSTRACT*** |  |  |  |
|  | 2 | Summary of study design, methods, results, and conclusions | N/A |
| ***INTRODUCTION*** |  |  |  |
|  | 3 | Scientific and/or clinical background, including the intended use and role of the AI approach | N/A |
|  | 4 | Study aims, objectives, and hypotheses | N/A |
| ***METHODS*** |  |  |  |
| ***Study Design*** | 5 | Prospective or retrospective study | FLOW AND TIMING |
|  | 6 | Study goal | N/A |
| ***Data*** | 7 | Data sources | PATIENT SELECTION |
|  | 8 | Inclusion and exclusion criteria | PATIENT SELECTION |
|  | 9 | Data pre-processing | N/A |
|  | 10 | Selection of data subsets | PATIENT SELECTION |
|  | 11 | De-identification methods | N/A |
|  | 12 | How missing data were handled | PATIENT SELECTION |
|  | 13 | Image acquisition protocol | INDEX TEST |
| ***Reference Standard*** | 14 | Definition of method(s) used to obtain reference standard | REFERENCE STANDARD |
|  | 15 | Rationale for choosing the reference standard | REFERENCE STANDARD |
|  | 16 | Source of reference standard annotations | REFERENCE STANDARD |
|  | 17 | Annotation of test set | REFERENCE STANDARD |
|  | 18 | Measures of inter- and intra-rater variability of features described by the annotators | REFERENCE STANDARD |
| ***Data Partitions*** | 19 | How data were assigned to partitions | FLOW AND TIMING |
|  | 20 | Level at which partitions are disjoint | FLOW AND TIMING |
| ***Testing Data*** | 21 | Intended sample size | PATIENT SELECTION |
| ***Model*** | 22 | Detailed description of model | INDEX TEST |
|  | 23 | Software libraries, frameworks, and packages | INDEX TEST |
|  | 24 | Initialization of model parameters | INDEX TEST |
| ***Training*** | 25 | Details of training approach | N/A |
|  | 26 | Method of selecting the final model | N/A |
|  | 27 | Ensembling techniques | N/A |
| ***Evaluation*** | 28 | Metrics of model performance | INDEX TEST |
|  | 29 | Statistical measures of significance and uncertainty | INDEX TEST |
|  | 30 | Robustness or sensitivity analysis | INDEX TEST |
|  | 31 | Methods for explainability or interpretability | N/A |
|  | 32 | Evaluation on internal data | INDEX TEST |
|  | 33 | Testing on external data | INDEX TEST |
|  | 34 | Clinical trial registration | N/A |
| ***RESULTS*** |  |  |  |
| ***Data*** | 35 | Numbers of patients or examinations included and excluded | PATIENT SELECTION |
|  | 36 | Demographic and clinical characteristics of cases in each partition | PATIENT SELECTION |
| ***Model performance*** | 37 | Performance metrics and measures of statistical uncertainty | INDEX TEST |
|  | 38 | Estimates of diagnostic performance and their precision | INDEX TEST |
|  | 39 | Failure analysis of incorrect results | INDEX TEST |
| ***DISCUSSION*** |  |  |  |
|  | 40 | Study limitations | N/A |
|  | 41 | Implications for practice, including intended use and/or clinical role | N/A |
| ***OTHER INFORMATION*** |  |  |  |
|  | 42 | Provide a reference to the full study protocol or to additional technical details | N/A |
|  | 43 | Statement about the availability of software, trained model, and/or data | N/A |
|  | 44 | Sources of funding and other support; role of funders | N/A |

# Comprehensive definitions of extracted performance metrics for detection and segmentation of nasopharynx lesion

## Intersection-over-union (IoU)

IoU is a common evaluation metric used in computer vision, particularly in object detection tasks. It quantifies the overlap between two bounding boxes, i.e., the predicted bounding box and the ground truth bounding box, and provide an objective assessment of the detection accuracy. More specifically, IoU is calculated as the area of overlap between the two boxes (typically 2D) divided by the area of their union. The IoU is also known as the Jaccard index, but the later name is rarely used in the context of object detection and more frequently used in segmentation. The formal definition is:

$$\mathrm{IoU}\left( A,B \right)=\frac{\left| A\cap B \right|}{|A\cup B|}$$

where A and B are the area enclosed by the predicted and ground-truth bounding box, respectively. The result remains the same when A and B are switched.

## Dice similarity score (DSC)

Similar to IoU, DSC also provide a quantitative and objective measure of the quality of a prediction based on it’s overlapping in area/volume with the ground-truth. However, DSC is typically more commonly reported as a measure to assess the medical imaging segmentation precision then IoU. One key difference between IoU and DSC is the stress towards sensitivity, especially in presence of small structures; it is generally more forgiving of small mismatches but maintain penalization of false-positive predictions. Additionally, it emphasizes overlap while accounting for the object’s size, making it particularly useful in scenarios where the objects being measured can vary significantly in size. The formal definition is:

$$\mathrm{DSC}\left( A,B \right)=\frac{2\left| A\cap B \right|}{\left| A \right|+|B|}$$

where definitions A and B remain the same as above. Same as IoU, the results are the same when A and B are switched.

## Average surface distance (ASD)

The definition of ASD is straight-forward, it is the distances between the two closest points on the two referenced segmentation, averaged across the entirety of the two surfaces. Unlike DSC, ASD is a dissimilarity metric that measures the discrepancy between two segmentations. ASD is an important metric that complements DSC because areal or volumetric overlap can under-represent the complexity of the objects’ morphology. For instance, if a highly convoluted structure but overall spherical target (e.g., a kidney) is delineated with a spherical segmentation, the DSC will be very high because majority of the volume is covered by the segmentation. However, the segmentation might not be considered accurate as the outline does not depict the convoluted structure properly. This limitation of DSC underscores the need for a complementary metric such as ASD to provide a better understanding of the segmentation performance of AI, especially when early NPC and benign changes such as hyperplasia can be complex in morphology. This is the key reason we take into consideration this extra metric in this systematic review. The formal definition of ASD is as follows:

$$ASD\left( S, S^{'} \right)=\frac{1}{n_{S}+n_{S^{'}}}\left[ \sum_{p=1}^{n_{s}} d\left( p,S^{'} \right)+\sum_{p^{'}=1}^{n_{S^{'}}} d\left( p^{'}, S \right) \right]$$

where $S$ and $S^{'}$ are the predicted and ground-truth segmentation surface, respectively, $n_{i}$ is the number of points evaluated for the i-th surface, $d(p,S)$ is the distance between point $p$ and surface $S$.

# Detailed applicability concerns evaluation

## Rationales of criteria checklist

This systematic review evaluates artificial intelligence (AI) studies within the literature for their applicability to MRI-based nasopharyngeal carcinoma (NPC) screening. Given the intended scopes of the reviewed studies was often not NPC screening, we design a checklist for evaluation of applicability concerns to facilitate reproducibility of this systematic review. In particular, only the “Patient Selection” domain remained relevant because both “Index Test” and “Reference Standard” domain are linked to the studies’ original purposes rather than NPC screening.

To be considered applicable for NPC screening, studies must include patients with stage I NPC (stage T1, N0, M0), which is the key group of patients to detect as they are the most challenging group to existing clinical workflows. Furthermore, an ideal study would reflect the typical demographic characteristics of an NPC screening program, maintaining a ratio of early stages (I/II) to advanced stages (III/IV) at approximately 7:3, as reported in previous NPC screening research^2^. Additionally, inclusion of non-NPC patients, such as those with benign lesions like hyperplasia, which are difficult to distinguish from early-stage NPC on MRI, is also crucial for applicability because many participants in a screening program will not have NPC but other abnormalities at the nasopharynx.

Additionally, the MRI imaging protocol of the selected patients is also crucial for determining applicability for NPC screening. Specifically, applicable AI algorithm should be developed based on an imaging protocol that does not require the administration of intravenous-injected contrast agents as they might introduce additional risks, scan time, as well as operational costs in the low-risk patients typically encountered in screening. Furthermore, the scanning protocol should also be quick using as few sequences as possible.

## Workflow for applicability evaluation

Items defined in the applicability checklists were evaluated for each included artificial intelligent (AI) studies. The statuses of the checklist items (in main text) were determined to be either “Yes”, “No” or “Unclear” based on a full-main-text and supplementary material examination conducted manually, with “Unclear” indicating the article did not report sufficient detail for the item.

Based on the evaluation results of the checklist, two reviewers (**BLINDED**) rated independently the studies into either “low”, “medium” or “high” applicability for screening nasopharyngeal carcinoma on MRI. The rating takes into consideration the tasks (direct detection, segmentation and/or discrimination) aimed to be performed by the AI. Differences in rating are resolved by a third reviewer, who is a board-certified radiologist subspecialized in head and neck radiology.

# Supplementary Results

## Sensitivity Tests

### Segmentation Studies

Figure S1	Leave-one-out sensitivity analysis of the pooled AI primary NPC segmentation performance in terms of (*Top*) Dice similarity score (DSC) and (*Bottom*) average surface distance (ASD). The forest plot shows the pooled performances, their confidence range, and the heterogeneity measures $\boldsymbol{Q}$ statistics, $\boldsymbol{I}^{\boldsymbol{2}}$, and $\boldsymbol{\tau}^{\boldsymbol{2}}$ with the study labelled as row header excluded from the meta regression. For DSC, similar pooled results across studies were observed. But for ASD, one study (X Luo et al., 2023) showed apparent deviation from the rest of the studies. The “Overall” row shows the pooling without exclusion of outlier study.

### Discrimination Studies

Figure S2	Leave-one-out sensitivity analysis of the pooled AI primary NPC discrimination performance (between NPC and non-NPC). Radiomics studies (LM Wong 2022 & J Cheng 2024) were the main contributors of heterogeneity. Therefore they were excluded from the analysis. Here we only showed the I^2^ because the bivariate meta-regression was conducted in log-transformed space, making the interpretation of Q statistics and $\boldsymbol{\tau}^{\boldsymbol{2}}$ difficult.

| **Scenario** | **k** | **Sens** | **Sens [95% CI]** | **Spec** | **Spec [95% CI]** |
| --- | --- | --- | --- | --- | --- |
| Baseline (all 6) | 6 | 0.971 | [0.880, 0.994] | 0.878 | [0.789, 0.932] |
| Keep Ke 2020 + Wong 2021 | 4 | 0.972 | [0.908, 0.992] | 0.877 | [0.835, 0.909] |
| Keep Ke 2020 + Wong 2022 | 4 | 0.953 | [0.790, 0.991] | 0.852 | [0.743, 0.919] |
| Keep Deng 2022 + Wong 2021 | 4 | 0.975 | [0.882, 0.995] | 0.885 | [0.809, 0.933] |
| Keep Deng 2022 + Wong 2022 | 4 | 0.964 | [0.729, 0.996] | 0.873 | [0.698, 0.954] |

Table S3	Pairwise drop sensitivity anlaysis to investigate the potential influences of potential overlap in patient cohorts for discrimination studies. Se = sensitivity, Sp = specificity, CI = confidence interval.

# Reference (Supplementary)

1.	Mongan J, Moy L, Kahn Jr CE. Checklist for artificial intelligence in medical imaging (CLAIM): a guide for authors and reviewers. Radiological Society of North America; 2020:e200029.

2.	Chan KCA, Woo JKS, King A, et al. Analysis of Plasma Epstein-Barr Virus DNA to Screen for Nasopharyngeal Cancer. N Engl J Med 2017;377(6):513-522. DOI: 10.1056/NEJMoa1701717.
